# CRISPR-Cas systems target a diverse collection of invasive mobile genetic elements in human microbiomes

**DOI:** 10.1186/gb-2013-14-4-r40

**Published:** 2013-04-29

**Authors:** Quan Zhang, Mina Rho, Haixu Tang, Thomas G Doak, Yuzhen Ye

**Affiliations:** 1 School of Informatics and Computing, Indiana University, 150 South Woodlawn Avenue, Bloomington, IN 47405, USA; 2 Department of Biostatistics and Bioinformatics, Roswell Park Cancer Institute, Elm & Carlton Streets, Buffalo, NY 14263, USA; 3 Center for Genomics and Bioinformatics, School of Informatics and Computing, Indiana University, 150 South Woodlawn Avenue, Bloomington, IN 47405, USA; 4 Department of Biology, Indiana University, 1001 East Third Street, Bloomington, IN 47405, USA

**Keywords:** CRISPR-Cas system, human microbiome, mobile genetic element (MGE)

## Abstract

**Background:**

Bacteria and archaea develop immunity against invading genomes by incorporating pieces of the invaders' sequences, called spacers, into a clustered regularly interspaced short palindromic repeats (CRISPR) locus between repeats, forming arrays of repeat-spacer units. When spacers are expressed, they direct CRISPR-associated (Cas) proteins to silence complementary invading DNA. In order to characterize the invaders of human microbiomes, we use spacers from CRISPR arrays that we had previously assembled from shotgun metagenomic datasets, and identify contigs that contain these spacers' targets.

**Results:**

We discover 95,000 contigs that are putative invasive mobile genetic elements, some targeted by hundreds of CRISPR spacers. We find that oral sites in healthy human populations have a much greater variety of mobile genetic elements than stool samples. Mobile genetic elements carry genes encoding diverse functions: only 7% of the mobile genetic elements are similar to known phages or plasmids, although a much greater proportion contain phage- or plasmid-related genes. A small number of contigs share similarity with known integrative and conjugative elements, providing the first examples of CRISPR defenses against this class of element. We provide detailed analyses of a few large mobile genetic elements of various types, and a relative abundance analysis of mobile genetic elements and putative hosts, exploring the dynamic activities of mobile genetic elements in human microbiomes. A joint analysis of mobile genetic elements and CRISPRs shows that protospacer-adjacent motifs drive their interaction network; however, some CRISPR-Cas systems target mobile genetic elements lacking motifs.

**Conclusions:**

We identify a large collection of invasive mobile genetic elements in human microbiomes, an important resource for further study of the interaction between the CRISPR-Cas immune system and invaders.

## Background

Bacterial genomes are by no means static - they constantly exchange genetic materials, primarily through the action of various types of mobile genetic elements (MGEs). Horizontal transfer of MGEs is a key driving force in bacterial evolution, allowing bacteria to rapidly develop new traits. Many human pathogens acquire strain-specific properties and functions through foreign DNAs delivered by bacteriophages and plasmids - important factors in the spread of antibiotic resistance [1-4]. Another class of MGEs is integrative and conjugative elements (ICEs), bacterial MGEs that primarily reside in the host cell's chromosome, yet have the ability to transfer between cells by conjugation. But unlike plasmids, ICEs cannot be maintained in an extrachromosomal state because they cannot replicate autonomously, although this is still under investigation [5]. There are also genomic islands, a more general term for any cluster of genes in bacterial genomes acquired from horizontal transfers; an island can be a 'pathogenicity island' or a 'metabolic island' - among others - according to the functions of its genes [6,7].

Bacteria have developed various defense systems to limit the exchange of MGEs. Bacterial innate immunity is achieved by adsorption-blocking, methylation-restriction systems, and production of extracellular matrix, among other mechanisms [8], whereas adaptive immunity systems acquire invasive DNAs and use them for interference against further invasion of matching foreign DNA molecules. The clustered regularly interspaced short palindromic repeats (CRISPR)-CRISPR-associated (Cas) proteins systems are an RNA-guided adaptive immunity system that provides sequence-directed defense against MGEs [8-14]. CRISPR-Cas systems are found in most archaeal and some bacterial genomes [9,15] (see a list of the genomes at the CRISPRdb website [16]). Recently, a bacterial type II CRISPR-Cas system (which uses cas9) has been engineered to achieve guided genome engineering in human cells [17,18], *Saccharomyces cerevisiae *[19] and Zebrafish embryos [20], and to achieve selective repression of gene expression in *Escherichia coli *(by using a catalytically dead Cas9 lacking endonuclease activity) [21].

In general, CRISPR spacer-repeat arrays consist of 24 to 47 bp direct repeats flanking unique spacers acquired from foreign DNAs that have invaded the host and been stored in CRISPR arrays as a consequence (the donor sequences for spacers in the MGEs are called protospacers). To affect interference, these arrays are transcribed as precursor RNAs, and subsequently truncated to short CRISPR RNAs by Cas proteins encoded next to the CRISPR array, and used to guide subsequent attacks at the protospacers on matching invaders [22,23]. A consequence of the constant arms race between bacteria and invading DNA sequences (via CRISPR-Cas systems) is the rapid turnover of the spacers in CRISPR arrays. For example, an analysis of the streptococcal CRISPRs from human saliva, in which CRISPR spacers and repeats were amplified from salivary DNA - using the conserved streptococcal CRISPR repeat sequence for priming - revealed substantial spacer sequence diversity within and between subjects over time [24].

Most previous studies of CRISPR-Cas defense systems have focused, not surprisingly, on their interactions with phages [25-28], with fewer studies of other types of MGEs that may be defended against by CRISPR defense systems [10]. Considering that viruses are the most abundant organisms on earth, our understanding of viral diversity, functions and interactions is still very limited, and understanding the interactions between bacterial hosts and viruses is an essential step toward understanding bacterial ecology. As a practical application, the warfare between bacteriophage and host via CRISPR defense systems in dairy fermentation processes has been extensively studied (for example, *Streptococcus thermophiles*, which utilizes CRISPRs to defend against its active invaders, *S. thermophiles *bacteriophages [10,23]).

Fewer studies have examined interactions between CRISPR-Cas systems and plasmids. Marraffini and Sontheimer [2] discovered that a clinical isolate of *Staphylococcus epidermidis *contains a spacer that is homologous to the *nickase *gene found in most staphylococcal plasmids, and showed that CRISPR interference prevents conjugation and plasmid transformation in *S. epidermidis *(at the DNA level), and the interference could be blocked by insertion of a self-splicing intron into *nickase at the *protospacer site. Palmer and Gilmore identified matches between CRISPR spacers found in 16 *Enterococcus faecalis *draft genome sequences and sequences from mobile elements, including pheromone-responsive plasmids and phage, and found a highly significant inverse correlation between the presence of a CRISPR locus and acquired antibiotic resistance in *E. faecalis*, suggesting that antibiotic use inadvertently selects for enterococcal strains with compromised genome defense and an increased ability to acquire drug-resistance-encoding plasmids [4].

Bacteria and phage (and other types of MGEs) co-exist and co-evolve in diverse environments, and a better understanding of the interactive relationship between host and MGEs requires a metagenomic approach. Most metagenomic studies [29-31] focus on only one side of the story: either the bacteria or the MGEs. Sequencing of viral-like particles enriched from fecal samples obtained from healthy adult female monozygotic twins (co-twins) and their mothers revealed high inter-personal diversity even among co-twins and their mothers, whereas temporal intra-personal variation in viromes is very low [32]. Similarly, pyrosequencing of virus-enriched metagenomes isolated from bovine rumen fluid has identified up to 28,000 different viral genotypes, revealing a high viral diversity in the rumen microbiome [30]. Combined analysis of CRISPR-Cas systems and viruses can be more revealing. For example, Pride *et al*. [33] compared their collection of streptococcal CRISPR sequences (derived by a targeted sequencing approach [24]) with virome reads in the saliva of four human volunters over 17 months, and observed co-existence of spacers and viruses, suggesting that streptococcal CRISPR-Cas systems are under constant pressure from salivary viruses.

Andersson and Banfield [29] reconstructed virus and host eubacterial and archaeal genome sequences from community genomic data from two natural acidophilic biofilms, and matched viruses with their hosts using spacers. Although acidophilic biofilms are rather simple microbial communities, this work demonstrated that by extracting both bacterial and viral sequences, and identifying their interactions through CRISPRs, metagenomics can become a powerful tool to reveal fundamental rules governing the microbial world: for example, Andersson and Banfield suggest that community stability is achieved by rapid but compensatory shifts in host resistance levels and virus population structure. A more recent study utilizes spacer-protospacer relationships to discover potential viral contigs in much more complex microbial communities, human gut microbiomes [34], pioneering the use of CRISPR spacers to recover MGE sequences from microbial community sequencing data, and opening up opportunities for studying the arms race between viruses and their bacterial hosts.

As a more comprehensive approach to understanding the interactions between bacterial hosts and invading DNAs (not limited to phages) in human microbiomes, we have investigated about 700 human microbiome datasets, collected by the Human Microbiome Project (HMP) consortium [35,36]. This study has allowed us to identify novel MGEs that are involved in CRISPR-Cas systems and characterize their properties. Our discovery of MGEs is leveraged on our previous systematic annotation of CRISPR repeat-spacer arrays by targeted assembly, which first pools reads that contain repeats and then assembles the pooled reads into CRISPR arrays with repeat-spacer units [37]. We analyzed all the non-redundant candidate MGEs targeted by multiple spacers. Analysis of these MGEs demonstrates that the CRISPR-Cas defense systems target not only phage, but also other types of MGEs, including plasmids, genomic islands and ICEs. Notably, we report the first CRISPR-Cas systems targeting ICEs; a previous study [3] showed that a CRISPR system inhibits prophage acquisition in *Streptococcus pyogenes*, but in spite of the presence of 12 ICEs distributed in 13 sequenced *S. pyogenes *strains, no spacers were found that were acquired from these ICEs. A joint analysis of the CRISPR arrays and MGEs allows us to study the interaction patterns between the bacterial CRISPR systems and invaders. We observed that protospacer-adjacent motifs (PAMs) drive the resistance network between MGEs and CRISPR systems, with exceptions - some CRISPR systems target MGEs lacking the classical PAM sequences or any other conserved motif.

## Results

### Identification of CRISPR-targeting MGEs in human microbiomes

We identified 95,052 contigs or scaffolds (in the paper we call both 'contigs' for simplicity) that are candidate MGEs with evidence of CRISPR-Cas defenses. We applied CD-HIT-EST [38] to remove contig redundancy (using 80% sequence identity as the cutoff), resulting in 20,504 non-redundant MGE contigs (this collection is referred as MGE_nr). We note that these numbers are conservative estimates, as we applied stringent criteria in defining putative MGEs (for example, we required that a candidate MGE contig contain at least three protospacers, since some CRISPR-Cas systems contain self-targeting spacers [39]; by contrast, Stern *et al*. only required a contig to contain one protospacer [34]), and generous criteria in eliminating redundancy (see Methods for details). We also prepared a smaller collection of MGEs (referred to as MGE_sel) with 959 large MGE segments (of at least 5 kbp), each segment containing at least 40 protospacers. MGE_nr and MGE_sel were used in our further analyses.

**Figure 1:**
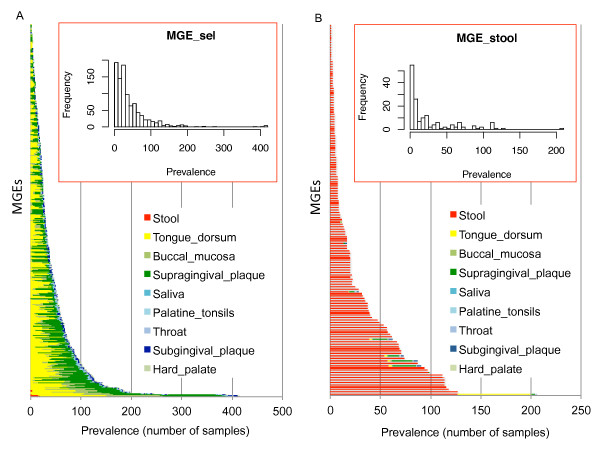
**The distribution of MGEs across HMP samples**. A MGE is considered to be present in a sample if 70% of its length is covered by at least one read. The X-axis represents the number of samples that contain each MGE, Y-axis represents MGEs. **(A) **Distribution of the 959 MGEs each with at least 40 protospacers. **(B) **Prevalence of 155 MGEs selected from stool microbiomes, each with at least 10 protospacers. The inserts are the histograms of the MGE sizes. MGE, mobile genetic element.

MGE_nr contains many large contigs (see Figure 1 in Additional file 1 for a histogram of contig sizes). In total there are 253 contigs longer than 100 kbp; by contrast, a majority of phages have genomes smaller than 100 kbp and average 40 kbp (see Figure S2 in Additional file 1). The actual MGEs may in some cases be shorter, as contigs can contain parts of the flanking bacterial genomes, but there are still four MGE segments with dense protospacers (see Methods) covering more than 100 kbp, including: SRS050669_LANL_scaffold_47865 containing 250 protospacers between 855 and 159,934 bp; SRS022530_LANL_scaffold_21325 with 194 protospacers between 2,493 and 123,573 bp; SRS062761_LANL_scaffold_30103 with 256 protospacers between 216 and 118,723 bp; and SRS051791_LANL_scaffold_3222 with 61 protospacers between 3,062 and 116,815 bp (Figure S1 in Additional file 1 shows the histogram of the segment sizes). As the HMP datasets contain longer reads than the MetaHit datasets, we were able to identify longer MGEs than the MGEs discovered in [34]. For example, three phages identified from MetaHit contigs (scaffold53395_2_MH0022, scaffold54386_1_MH0053 and scaffold4504_1_O2.UC-4) [34] match a single MGE contig we identified in a stool sample (SRS014459_WUGC_scaffold_93237 of 34,292 bp) with >99.5% sequence identity. These three MetaHit contigs have significantly non-overlapping regions (and they match to 67 to 11,764, 2,678 to 24,468, and 16,706 to 33,639 bp, respectively, in the HMP contig), and therefore were treated as three different phages in [34]. This comparison indicates that we will be able to refine our discovery of novel phages (and other MGEs) as better metagenome assemblies become available - for example, due to the use of longer reads.

### Oral sites carry a greater variety of MGEs

In healthy human populations, oral and stool bacterial communities are especially diverse - and have similar diversity - in community membership, as shown in [35]. However, our study suggests that oral sites have a much greater variety of phages and other MGEs, as shown in the body site breakdown of the source samples from which MGEs were identified (Table 1): 1,206 MGE contigs (with a total of 36,731,315 bases) were identified from 150 stool samples (on average each sample has a total of 104,115,088 bases); by contrast, 13,017 MGE contigs (with a total of 127,112,013 bases) were identified from 136 tongue dorsum samples, each containing an average of 85,590,125 bases.

**Table 1:** The breakdown of identified MGE contigs (the MGE_nr collection) by body site

Body site	Total samples	Average sample size (bp)	Total MGE contigs	Total MGE bps
Stool	150	104,115,088	1,206	36,731,315
Tongue dorsum	136	85,590,125	10,317	127,112,013
Supragingival plaque	129	57,371,899	6,619	72,553,937
Buccal mucosa	122	13,427,236	1,746	16,583,285
Palatine tonsils	6	22,409,573	141	2,358,527
Posterior fornix	60	7,715,463	50	835,115
Anterior nares	94	1,746,191	14	177,366

bp, base pair; MGE, mobile genetic element.

We examined the prevalence of the 959 MGE_sel blocks (see Methods) across the samples by reads recruitment. As shown in Figure 1A, some MGEs are prevalent (present in more than half of the samples), while others are only sparsely found in the healthy human population (see the insert in Figure 1A for the histogram of the MGE prevalence). Since the MGE_sel set includes very few MGEs prevalent in stool samples, we examined a set of 155 MGE segments selected from stool samples, each containing at least 10 (instead of 40) protospacers and found that the general observation holds - some MGEs are prevalent while others are sparsely distributed (see Figure 1B). Figure 1 also shows that MGEs are largely body site-specific: MGEs are prevalent either in oral sites, or in stool samples, but not in both. Although different oral sub-sites tend to share MGEs (for example, the prevalence profiles of MGEs across buccal mucosa and supragingival plaque samples are highly correlated, with Pearson correlation coefficient of 0.61; *P *<2.2e^-16^), we observed MGEs with oral sub-site preference. Examples include MGE contig SRS064423_LANL_scaffold_55363, which is widely distributed in tongue dorsum (108 samples) and supragingival plaque samples (67), but only in 7 buccal mucosa samples; SRS016002_WUGC_scaffold_521 found in most tongue dorsum datasets (118), but only in a few buccal mucosa (3) and supragingival plaque (3) samples; and SRS016575_Baylor_scaffold_2114 found mostly in supragingival plaque samples (113).

The body site-specific distribution of MGEs is also reflected by the constrained distribution of spacers in the HMP samples: 8,871 contigs (43%) match spacers found only in one body site; the remaining contigs have matching spacers found in multiple body sites (mostly different oral sites). In rare cases (114 contigs; 0.5%), contigs match spacers found in both stool and non-stool samples. SRS018443_Baylor_scaffold_7069 (assembled from a buccal mucosa sample) is a special case, with spacers found in CRISPRs from most body sites, including supragingival plaque, tongue dorsum, buccal mucosa, attached or keratinized gingiva, stool and throat. This contig is 57,301 bp, with 174 spacers distributed across almost the entire contig (566 to 57,111 bp); it represents a potential phage infecting streptococcus, sharing 73% sequence identity with *Streptococcus *phage Dp-1 (GI:327198314; which has a linear DNA genome of 56,506 bp).

### Annotation of MGEs

Similarity searches of the 20,504 MGE contigs in MGE_nr against known phage and plasmid genomes show that a surprisingly small proportion of the contigs are homologous to known MGEs, even when loose criteria are applied (sequence identity >50% and coverage >50% of the contig or the reference genome): 901 contigs (4.4%) are similar to known phage genomes, 402 contigs (2.0%) are similar to known plasmid genomes, and 110 (0.5%) are similar to known ICEs.

Among the 901 contigs with similarity to known phage genomes, 844 contigs can be assigned to specific viral families according to the International Committee on Taxonomy of Viruses classification [40]: 632 (75%) Siphoviridae; 173 (20%) Myoviridae; and 39 (5%) Podoviridae. Although our MGEs come from many different body sites (supragingival plaque, tongue dorsum, buccal mucosa, attached or keratinized gingiva, stool and throat), these ratios are surprisingly close to those inferred from gut-only samples (78%, 11.5% and 6.5%) [33], indicating that similar types of viruses live in these different environmental niches. We did not find any additional classes of known viruses in the HMP metagenomic datasets.

Although only a small proportion of the MGEs show extensive similarity to known phage or plasmid genomes and ICEs - suggesting that the contigs represent intact phage or prophage - many more MGEs carry phage- or plasmid-related genes. A total of 6,820 MGE contigs (33.3%) contain one or more of the virus or plasmid proteins collected in the ACLAME database (version 0.4) [41]. In particular, capsid and tail proteins for bacteriophage structure and recombination-related proteins were predicted in most of these contigs. Annotation based on Basic Local Alignment Search Tool (BLAST) searches against the nr database (in which annotations are extracted from the descriptions of the sequences found by the BLAST searches) found a larger proportion of MGE contigs containing plasmid- or phage-related genes (14,071 contigs; 68.6%). Still, there are 6,433 (31.4%) contigs that lack any known plasmid- or phage-related genes, and the identities of these MGEs remain to be established.

To study the functional distribution of MGEs we used the MGE_sel collection, to minimize contamination by flanking host genomes. There are 30,904 protein-coding genes predicted from the spacer-targeted segments of the 959 MGE_sel set. A total of 10,664 proteins could be assigned to a clusters of orthologous groups (COG) family, but only 5,689 were assigned to well-characterized COG families (that is, excluding the two categories general function [R] and functional unknown [S]). Consistent with previous results, the most abundant known functions are related with replication, recombination and repair; and the second most abundant functions are related with transcription (see the functional distribution in Figure S3 in Additional file 1). We also summarized the Gene Ontology (GO) annotations of the genes (downloaded from the DACC website) [42], and analyzed the GO terms enriched or depleted in these MGE contigs, using binomial tests with a Bonferroni multiple testing correction. Not surprisingly, GO:0006260 (DNA replication), GO:0000746 (conjugation), GO:0042742 (defense response to bacterium) and GO:0005198 (structural molecule activity, associated with viral structural proteins) are among the 76 GO terms that appear significantly enriched among the annotations associated with MGEs. Example GO terms that are significantly depleted include GO:0042626 (ATPase activity, coupled to transmembrane movement of substances), GO:0016491 (oxidoreductase activity) and GO:0046872 (metal ion binding). Additional file 2 lists significantly enriched (73) or depleted (76) GO terms. We also checked specific functions, including bacterial restriction-modification systems. Among the 10,664 proteins, 141 proteins show similarity to proteins that are annotated as associated with bacterial restriction-modification systems (types I, II, and III) (see Additional file 3 for a list of these proteins). For example, a gene in MGE SRS015215_WUGC_scaffold_293, located between 25,616 and 27,199 bp, encodes a protein that is annotated as a type III restriction enzyme, res subunit. A similarity search of this protein against the PFAM database (see Methods) shows that this protein contains two domains: ResIII (type III restriction enzyme, res subunit) with an E-value of 5.3e^-28^, and Helicase_C (Helicase conserved C-terminal domain) with an E-value of 3.5e^-11^.

### Demonstration of various types of MGEs

MGEs identified in this study can be traced to plasmids, phages, ICEs and genomic islands; however, many are still of unknown types (see above). Some MGEs are extremely long, as compared with known phages and plasmid genomes, and contain genes encoding proteins of various functions. In this section, we use a few examples to demonstrate the diversity of the catalog of MGEs identified in the human microbiome.

The longest MGE found in a contig (SRS053630_LANL_scaffold_8877) is 386,225 bp. In total nine protospacers were identified in this contig, spanning almost the entire length (373,639 bp between 6,172 and 379,810). Similarity searches against reference genomes show that this contig is similar to a region in the *Haemophilus parainfluenzae *T3T1 genome (NC_015964.1) with a sequence identity of 92.7%. No phage- or plasmid-specific genes are found in the contig through similarity searches against the nr database by BLAST. However, searches against the ACLAME database show that this contig contains many plasmid-related genes, suggesting this MGE is an integrated plasmid associated with the *H. parainfluenzae *species. This is an extremely long MGE with 371 protein-coding genes (within the region bounded by the protospacers in the contig), considering that the longest known bacterial phage genome is 316,674 bp and most plasmid genomes are shorter than 50 kbp (see Figure S2 in Additional file 1 for the length distributions of phages and plasmid genomes).

SRS022530_LANL_scaffold_56387 (of 276,838 bp) contains 201 protospacers, spanning 273,713 bp (between 2,503 and 276,215 bp). Similarity searches show that this contig contains a gene (located between 208,333 and 208,851 bp) encoding T4 lysozyme, indicating that it is a T4-like phage; but it does not share overall similarity with known T4-like phage genomes and known T4-like phages have smaller genomes (for example, Enterobacterial phage T4 has a genome of 168,903bp). There is a Ser-tRNA gene at the end of the contig between 275,367 and 275,280 bp, suggesting that this contig represents a prophage, inserted into its host's genome at a tRNA gene (a typical prophage insertion site). This contig contains a large number of protein-coding genes: 241 genes (based on FragGeneScan [43] prediction) for the entire contig, or 240 genes in the region with protospacers. These genes encode proteins of various functions. For example, a cluster of three genes (gene locations are 73,86 to 74,986 bp, 74,999 to 75,904 bp, and 76,005 to 76,973 bp) encodes proteins with activities involving Fe-S, including Fe-S oxidoreductase (COG0641 and COG0535).

**Figure 2:**
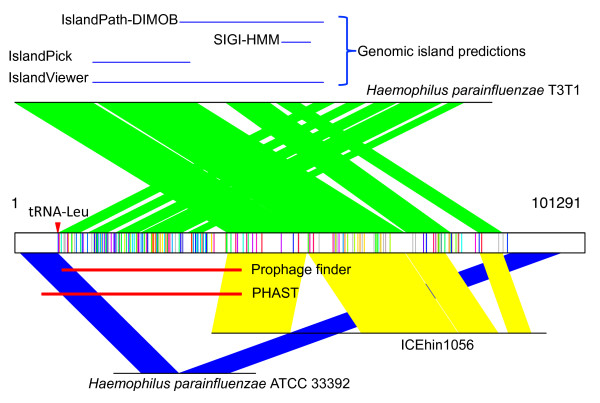
**MGE contig SRS016200_WUGC_scaffold_12207 represents a mosaic genomic island**. The contig is 101,291 bp long, and is shown as an open box in the middle, with protospacers shown as vertical lines within the box (gray lines each show a protospacer that is similar to spacers from individual HMP datasets, while lines of the same color show sets of protospacers matching spacers all from the same HMP dataset). Similarities between this contig and two bacterial genomes, *H. parainfluenzae *T3T1 (with sequence identity of 93%) and *H. parainfluenzae *ATCC 33392 (with sequence identity of 97%), and one ICE (ICEhin1056 found in *H. influenzae*), are represented as green, blue and yellow shades, respectively, between the contig and the corresponding genome lines. The location of the tRNA-Leu gene found in the contig is highlighted by a red triangle. The prophage regions found in this contig by Prophage finder and PHAST are shown as red lines, below the contig box. The regions corresponding to the genomic island regions in the *H. parainfluenzae *T3T1 genome as predicted by various methods (IslandViewer, IslandPick, SIGI-HMM and IslandPath-DIMOB) are represented as blue lines above the corresponding genome line.

SRS016200_WUGC_scaffold_12207 is likely to be a genomic island in a genome that is similar to the genomes of *H. parainfluenzae *strains (Figure 2). This contig is 101,291 bp. The MGE region in this contig is similar to the *H. parainfluenzae *T3T2 genome, whereas the two flanking ends are more similar (with sequence identity of 97%) to another *H. parainfluenzae *strain (*H. parainfluenzae *ATCC 33392), which apparently does not contain a similar MGE region, so that the two flanks are immediately adjacent in strain 33392. Similar to the genome of *H. parainfluenzae *T3T2, this contig is a mosaic genomic island, featuring two elements: one is a prophage (the prophage regions predicted by Prophage Finder and PHAST (see Methods) are shown in Figure 2), and the other is similar to ICEhin1056, an ICE found in *H. influenzae*. However, the two elements are assembled in the genomic island in a different order - there is a crossover of the similarities between the genomic islands in the contig and in the reference *H. parainfluenzae *T3T2 (see Figure 2). There are 201 protospacers spanning the two elements, indicating that they form a continuous MGE element (that is, a genomic island) in this contig. This example also illustrates that analysis based on protospacers can be employed to improve the annotation of genomic islands in complete genomes. Our analysis suggests that the actual genomic island in the genome of *H. parainfluenzae *T3T2 is larger than predictions by various programs, including IslandViewer [44], IslandPick [45], SIGI-HMM [46] and IslandPath-DIMOB [47]. We used IslandViewer server [48] for all the predictions of genomic island.

SRS024447_LANL_scaffold_50338 also contains an ICE, similar to the ICE Tn*GBS1 *found in *Streptococcus agalactiae*. TnGBS1 presents an atypical family of conjugative transposons named Tn*GBS*s, which associate DDE transposition and conjugation [49]. The comparison of Tn*GBS1 *and Tn*GBS2 *(another ICE found in *S. agalactiae *NEM316) shows that they encode only five homologous proteins, including the putative transposases, and three putative surface-exposed LPxTG proteins similar to surface exclusion proteins [49]. The contig we found in SRS024447 (a supragingival plaque sample) shares 70% overall sequence similarity with the Tn*GBS1 *found in strain NEM316, with a putative transposase gene (SRS024447.55152, located between 419 and 1,756 bp, sharing 66% sequence identity at the protein level with Gbs0410), and two genes (SRS024447.55167 located between 15,889 and 18,108 bp, and SRS024447.55168 located between 18,179 and 18,847 bp) that encode putative LPxTG proteins.

### Correlation study of MGEs and their bacterial hosts

We observed that some of the MGE contigs contain large regions without protospacers and are likely to be segments from the flanking bacterial host genomes. We utilized these contigs to study the correlation between the MGEs and their bacterial hosts.

**Figure 3:**
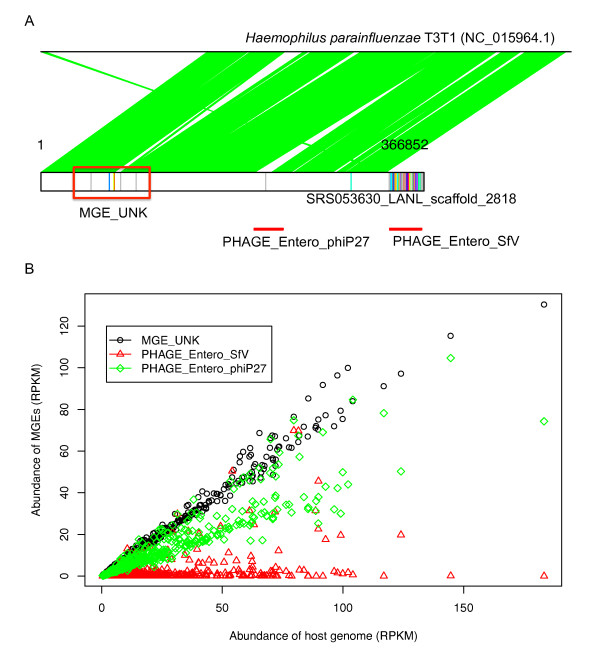
**SRS053630_LANL_scaffold_2818 contains three MGEs with differential abundance patterns across HMP samples**. **(A) **Annotation of the contig: the bottom box represents the contig (with protospacers shown as vertical lines), and the top line represents the genome of *H. parainfluenzae *T3T1, with which the contig shares similarity. Alignment between the genome and the contig is shown in green. This contig contains three MGEs, two of which are similar to phages based on PHAST predictions (the regions predicted to be prophages by PHAST are shown in red lines below the contig box), and one contains six protospacers (highlighted in the red box). The MGE similar to phage Entero SfV contains most of the protospacers. **(B) **Relative abundance of each MGE (Y-axis; MGE_UNK in open circles, PHAGE_Entero_SfV in red triangles, and PHAGE_Entero_philP27 in green diamonds) versus the abundance of bacterial host (X-axis) across the HMP samples. MGE, mobile genetic element; RPKM, reads per kbp per million reads.

One example is contig SRS053630_LANL_scaffold_2818 of 366,852 bp, which is assembled from a tongue dorsum sample. Combing the differential distributions of the protospacers along this contig and the PHAST [50] prediction of prophages, we predict that there are three MGEs of different invasion histories in this region of the bacterial genome (Figure 3A). Two MGEs are likely phages: one is similar to phage Entero SfV (based on PHAST prediction, and has the most protospacers (99 in total) as compared to the other two MGE regions); the other phage, which has only one protospacer, is similar to phage Entero phiP27. Another region (termed MGE_UNK, highlighted in the red box in Figure 3A) with six protospacers is likely to be a third MGE captured in this contig. Similarity searches show that this contig is similar to *H. parainfluenzae *T3T1, with a sequence identity of 93% covering almost the entire contig - excluding the MGE region similar to phage Entero SfV - suggesting that the MGE region annotated as phage Entero SfV is a more recent capture into this genome, close to a pre-existing MGE. This is further confirmed by abundance analysis of the three MGEs in human microbiomes: Figure 3B shows that the MGE of phage Entero SfV only co-occurs with the host bacterial genome (whose abundance was estimated using a large region between 91,271 and 204,149 bp in the contig that lacks any protospacers) in a few samples, indicating that in these samples, the MGE is integrated into the bacterial host. By contrast, the distribution of MGE_UNK is similar to the distribution of the bacterial host in all samples, indicating that this region has become part of the bacterial genome without active deletions.

**Figure 4:**
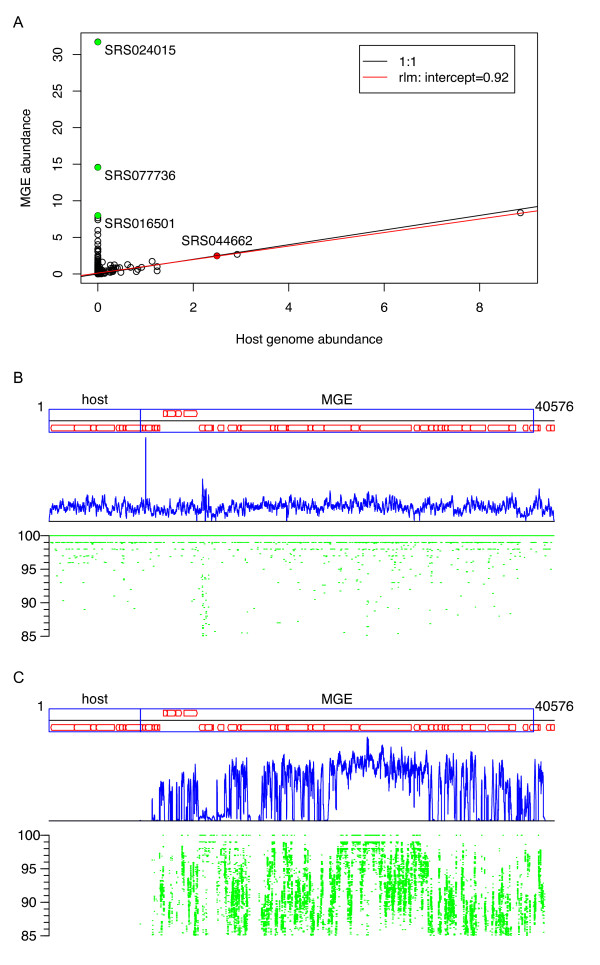
**Abundance analysis of the MGE found in SRS044662_LANL_scaffold_46036**. The MGE is predicted to be a prophage with genes encoding phage-related proteins (phage structural proteins, and so on). **(A) **Correlation between the abundance of the MGE and the abundance of its host, with the data points for 'outliers' in green, and the data point for the source sample (SRS044662) of this contig in red. **(B) **Detailed reads recruitment to the contig in sample SRS044662, from which the contig was assembled (green lines; the identity bar is shown on the left) and the coverage curve (in blue) along the contig. The genes predicted in this contig are shown as open arrows at the top (for the genes on the forward strand) and the bottom (for the genes on the reverse strand) of the line representing the contig. **(C) **Coverage and reads recruitment to the contig in another sample (SRS024015), demonstrating a case where MGE significantly outnumbers the host bacterium. MGE, mobile genetic element.

We carried out a large-scale correlation study between the MGEs and their bacterial hosts' distributions. From the MGE_sel collection, we identified 98 contigs that contain both MGEs and large segments (>5 kbp) of their bacterial host genomes (that is, without any protospacers). Most of these contigs can be classified into three categories based on linear fitting of the abundance of the MGE relative to the host genome, combined with manual checking of the abundance plots:

1. The abundance level of the MGE is very similar to its bacterial host in most samples, but in some samples significantly outnumbers the bacterial host, indicating either a burst of MGE replication in this bacterial host or a different - possibly additional - bacterial species hosting this MGE;

2. The MGE and the bacterial host are of similar abundances in the majority of the samples, indicating the MGE has became a 'permanent' resident in the corresponding host bacterial genome;

3. The abundance level of the MGE is significantly lower than its bacterial host in the majority of the samples, with occasional bursts of the MGEs in a few samples, suggesting recent invasions. Entero SfV found in SRS053630_LANL_scaffold_2818 (see above) belongs to category 3, while another MGE from the same contig (MGE_UNK) belongs to category 2. Figure 4 illustrates an example that falls into category 1: the relative abundant plot (Figure 4A) suggests that in most of the samples the MGE is of similar abundances to its bacterial host (see detailed reads recruitment in Figure 4B), but in some samples (for example, SRS024015), the MGE outnumbers the bacterial host (or the host is not detected at all), as confirmed by the read recruitment plot (Figure 4C). Table S1 in Additional file 1 shows a summarization of these MGEs based on their abundance curves.

### Protospacer-adjacent motif sequences are specific to CRISPR types - but some target MGEs lack protospacer-adjacent motifs

PAMs are conserved two- to three-nucleotides sequences, which appear immediately after or one nucleotide after protospacers [51] - thus, to be identified, spacers must be mapped to their targets as we have done on a metagenome scale here. PAMs are important for spacer acquisition into CRISPR arrays: they determine the targets of CRISPR-Cas defense systems (one reported exception is the CRISPR-Cas system found in *Yersinia pestis *[51]) and the orientation of spacers in the CRISPR arrays. For some CRISPR-Cas systems (type I and II), PAMs are also important for CRISPR RNA biogenesis [52]. CRISPR type-specific conservation has been observed in recent studies: known PAMs include CAT/CTT, GAA and GG [53].

**Figure 5:**
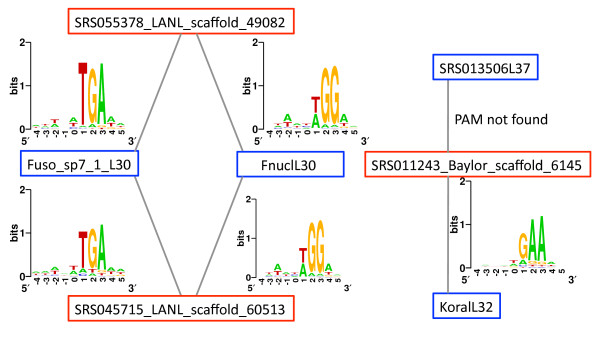
**A network of MGEs and CRISPR-Cas defense systems mediated through PAMs**. Only selected MGEs and CRISPR types are shown, for demonstration purposes. Blue rectangles represent CRISPRs; red rectangles represent MGE contigs. The WebLogo of PAM for the protospacers is plotted on the edges between the corresponding nodes of the MGE and CRISPR. The logos include 10 positions: -4 to 0 for the protospacers and 1 to 5 for the adjoining regions containing the PAM. PAM, protospacer-adjacent motifs.

Our large collection of MGEs and CRISPR arrays allows us to systematically study their interactions. We note that, in this study, we defined CRISPR types by their repeats' sequences in the CRISPR arrays as in [37]: such a classification schema was necessary because the complete CRISPR-Cas systems (with CRISPR locus and the *cas *genes) are often unavailable in metagenome assemblies; and it has been reported that repeat-based classification corresponds to a *cas *gene-based classification of CRISPR-Cas systems [54]. Our analyses show that, while most CRISPR types target multiple MGEs (unsurprising), there are also many cases where the same MGE is independently targeted by distinct CRISPR types. Figure 5 shows that the same CRISPR type utilizes similar PAMs when attacking various MGEs, whereas different CRISPRs attacking the same MGE acquire quite different spacers. For example, protospacers from SRS045715_LANL_scaffold_60513 are incorporated into arrays of three CRISPR types, utilizing two distinct PAMs (TGA for Fuso_sp7-1_L30, and T/AGG for FnuclL30 and FperiL30; see Figure 5).

We also observed that several CRISPR types attack MGEs lacking detectable PAMs, indicating that they rely on some cryptic motifs or a novel mechanism of spacer recognition, yet to be characterized. One notable case is SRS018394L37 (a novel CRISPR type discovered in the HMP sample SRS018394 [37], with repeat sequence GTATTGAAGGTTATCCATTTATAATAAGGTTTAAAAC). The MGE SRS022530_LANL_scaffold_21325 (a contig assembled from a tongue dorsum microbiome dataset) contains 251 protospacers, of which most (244) match spacers found in arrays of this particular CRISPR. For example, a protospacer located between 71,924 and 71,963 bp in the contig is identical to a spacer found in a large CRISPR array with 78 repeat-spacer units, which was assembled from another tongue dorsum microbiome dataset (SRS016319) by the targeted assembly approach [37]. But we could not detect any conserved short motif by aligning the adjacent regions of the 244 protospacers. Further, we were unable to detect any significant sequence motifs in either upstream or downstream adjacent regions using MEME (see Methods), a *de novo *motif detection tool that works independent of alignment. Another example is MGE SRS011243_Baylor_scaffold_6145. No motif can be detected in the adjacent regions of its protospacers that are targeted by CRISPR SRS013506L37; by contrast, its protospacers that are targeted by another CRISPR type (KoralL32) have a PAM of GAA as shown in Figure 5. Table S2 in Additional file 1 lists 13 CRISPR types that attack MGEs lacking PAMs.

Our joint analysis of CRISPRs and MGEs shows that there is a simple pattern governing the interaction network between the CRISPRs and MGEs: PAM sequences drive the interaction. However, powered by the large collection of metagenomic sequences (over 700 datasets), we were able to detect novel CRISPR systems in the human microbiomes, which attack MGEs without apparent PAM signals, suggesting that the interaction network might be more complex than it appears.

## Discussion

One potential application of CRISPRs is to improve the annotation of bacterial genomes. Many computational approaches have been developed to detect MGEs, using nucleotide composition differences between the MGEs and the host genomes [55] or comparative genomics approaches [56]. A limitation of the composition-based approaches is that nucleotide composition has large variation and it is not always sufficient for detecting alien genes (as shown in the various predictions of the genomic island in the *H. parainfluenzae *T3T1 genome; see Figure 2), while comparative genomics approaches rely on the availability of genomes. Discovery of MGEs *via *CRISPR-Cas systems provides an unbiased catalog of MGEs, independent of known homology and sequence composition biases - as evidenced by the extremely diverse collection we provide in this work. The traces of CRISPR-Cas defenses (the spacers) provide complementary information to sequence information (homology and composition), and we expect that a new breed of approaches will combine all this information to improve the annotation of alien regions and therefore of bacterial genomes.

Several questions arise from our analysis of MGEs. The first is that majority of the identified MGEs are not identified as phage, plasmids or ICEs, and so their mechanism of mobility is unknown. Second, MGEs carry genes that encode diverse functions, and a majority still have unknown functions. The third challenging problem is to discover possible mechanisms for those CRISPR-Cas systems that seem to not rely on the recognition of PAMs for spacer acquisition. A fourth problem is to identify the bacterial hosts of the MGEs - this task is difficult due to the fast mutation rates of MGEs and the high turnover rate of the spacers in CRISPR arrays. Attempts have been made - including our approach that uses contigs containing regions with protospacers (that is, integrated MGEs) and regions of flanking bacterial genomes (so both players were caught in the same contig), and a method that relies on spacer matching [34]; however, only a small proportion of the MGEs (or viruses) have had their putative bacterial hosts identified.

We observed an anti-correlation between the CRISPR-Cas systems and the presence of MGEs (data not shown), but we have also found many samples where MGEs and CRISPRs co-exist (also reported in [33]). We will look further into this phenomenon, and investigate the sequence variations of the MGEs in those samples, aiming to discover the possible mechanisms that MGEs adopt to escape CRISPR defenses (see [57]) in their natural environments.

We applied the common approach to detecting PAMs, by aligning the adjacent regions of predicted protospacers and then using Weblogo (see Methods) to detect motifs in the alignment. We checked both upstream and downstream regions of the protospacers, as the direction of the CRISPRs may be difficult to determine for some cases. Considering that this strategy may miss the motifs when the boundaries of the protospacers are not exactly defined, we also applied MEME to *de novo *detect the motifs in the adjacent regions. We cannot exclude completely the possibility that a PAM was not detected because mutations have occurred in many of the adjacent regions of protospacers, but given the large number of protospacers we could compare, we feel this is unlikely. In the future we will investigate those predicted MGEs that lack PAMs using additional approaches.

The same strategy of recovering MGEs using their traces in CRISPR-Cas systems can be applied to metagenomic datasets from other environments (for example, ocean, soil and other animal-associated environments). We foresee that, with a comprehensive resource of both CRISPRs and MGEs, we will be able to investigate the functionality of MGEomes and their impact on the structure and function of microbial communities.

## Conclusions

We have identified an extremely large collection of MGEs, using evidence of their having invading bacterial genomes and being defended against by CRISPR-Cas defense systems. Analysis of these MGEs demonstrates that the CRISPR defense systems target not only phage but also other types of MGEs, including plasmids, ICEs and genomic islands. A joint analysis of the CRISPR arrays and MGEs allows us to study the interaction patterns between the bacterial CRISPR-Cas systems and invaders. PAMs largely determine the resistance network: the same CRISPR type favors similar PAMs when attacking various MGEs, whereas different CRISPRs attacking the same MGE acquire quite different spacers. However, we observed that some CRISPR types target MGEs lacking classical PAM sequences or any other conserved motif, suggesting that the CRISPR resistance network might be more complex than it appears. We believe that this collection of MGEs will be a valuable resource to studies of the impacts of MGEs on microbial communities [58], and the arms race between bacteria and invasive DNAs (of various kinds).

## Methods

### Identification of MGEs using spacers

In our previous study, we developed a targeted assembly approach to assemble CRISPR arrays from shotgun metagenomic sequences [37]. The targeted assembly approach first pools reads that contain repeats found in known CRISPR-Cas systems or novel ones identified from whole metagenome assemblies, and then assembles the pooled reads to derive CRISPR arrays of repeats and spacers. Application of the targeted approach to the HMP datasets demonstrated a great improvement in the assembly of CRISPR arrays [37], and resulted in a large collection of 123,003 spacers (122,945 unique spacers).

HMP contigs containing multiple segments that share homology with spacers (protospacers) are predicted to be contigs containing invasive MGEs. BLAST searches were used to search the contigs in each sample against the collection of 122,945 unique spacers and 3,041 unique repeat sequences. We retained only contigs that contain three matched protospacers (which share at least 80% sequence similarity covering 80% of the corresponding spacers, with at least two extracted from CRISPR arrays of the same type), but no CRISPR repeats (>80% identities over 80% of the repeat sequence). As a result, we retained 95,052 contigs that are longer than 500 bps. This set is referred to as MGE_all.

### Subsets of MGE contigs

We further applied CD-HIT-EST [38] (80% sequence identity) to remove redundancy from the MGE candidate set, resulting in 20,504 contigs with a total of approximately 24 Mbp (this set is referred to as MGE_nr). Considering that some of the contigs may contain not only the MGEs but also parts of the host genomes (when MGEs are integrated into the bacterial host genome), and that it is important to minimize the contamination of the bacterial host genomes in some of the analyses - such as the statistical analysis of functional distribution of the MGE genes and the abundance analysis of the MGEs across different samples - we further prepared a collection of MGEs called MGE_sel containing 959 large segments (of least 5 kbp) from the MGE_nr contigs, which are densely populated with protospacers (at least 40 protospacers; and the maximum distance between two neighboring protospacers is 5 kbp). Our analyses focus on the two sets: MGE_nr and MGE_sel.

### Annotation of candidate MGE contigs

To characterize the identified MGEs, we carried out homology searches using BLAST against 690 phage genomes, downloaded from the National Center for Biotechnology Information ftp site [59] (selecting bacteria as the host); 1,156 plasmid genomes collected from the IMG database (version 3.5) [60]; and 466 ICEs downloaded from the ICEberg database (version 1.0) [61,62]. We consider that a contig is similar (or contains a region that is similar) to a phage, plasmid genome or ICE if they share similarity with sequence identity of >50%, covering >50% of the total length of either the phage, plasmid, ICE or the contig.

tRNAscan-SE-1.23 [63] and FragGeneScan [43] were used to predict tRNA genes and protein-coding genes, respectively. We predicted the functions of predicted proteins by similarity searches against multiple datasets of protein sequences and protein families, including the ACLAME database (which collects protein sequences of virus, plasmids, and prophages [64]), the National Center for Biotechnology Information nr database, PFAM families and COG families. The HMMs of the PFAMs (version 26.0) were downloaded from the ftp site [65]. For the COG families, we downloaded all protein sequences from the eggNOG v2.0 database [66] and retrieved the sequences with COG annotation [67]. MUSCLE [68] was used to generate a multiple alignment for each COG family, and the HMM builder from the HMMER3 package [69] was then applied to build a HMM for each COG. We built the HMMs of ACLAME families using a similar approach. For similarity searches against protein sequences (the nr database), we used RAPSearch2 [70] with an E-value cutoff of 0.001, and descriptions of the sequences are extracted to infer the functions of the query proteins. We used a keywords-based approach, and considered keywords 'phage', 'holin', 'tail', 'head' and 'capsid' for phage-related functions and 'plasmid', 'partition' and 'conjugative transfer' for plasmid-related functions. HMMER searches (by hmmscan from the HMMER3 package) were used to annotate the predicted proteins against protein families (PFAM, ACLAME and COG), with an E-value cutoff of 0.01.

We also used the gene annotations and their GO assignments for the HMP contigs available at the DACC website [42] for the enrichment analysis of the GO terms associated with the MGEs.

### Detecting protospacer-adjacent motif patterns

We used MGE contigs with at least 20 protospacers targeted by the same CRISPR type (some MGEs have protospacers targeted by different CRISPR types) for PAM analysis. Five base pair sequences in the adjacent regions (upstream and downstream regions are considered separately) of the protospacers and 5 bp sequences from the protospacers were extracted and aligned for conservation calculation and visualization. We calculated the conservation of each position in the alignment as the difference between the maximum possible entropy (2 bits) and the entropy of the observed base distribution as in [71]. We used Weblogo3.0 [72] for visualization of the motifs.

For the MGE contigs that do not contain typical PAMs (none of the adjacent positions has a conservation score of ≥0.5 bits), we also used MEME [73] for *de novo *predictions of motifs in the adjacent regions of the protospacers (including 2 bp from the protospacers, considering that the exact boundaries of the protospacers may not be identified, and 10 bp sequences preceding or after the protospacer regions).

### Detecting prophages

We searched MGE contigs against the prophage protein database ACLAME (version 0.4) [64] using BLASTX, which is the first step in Prophage Finder [74]. Subsequently, we ran Prophage Finder [75], with the BLASTX results as an input, to find potential prophage loci in MGE contigs. We also used the PHAST web sever [76] to predict prophages for selected examples.

### Quantification of MGEs

We used BLASTN to recruit reads to the MGE contigs. A read is considered to match a contig if the identity is at least 85%, covering 80% of the read. Abundances of the MGE contigs (the entire contig or segments of the contig) were calculated as reads per kbp per million reads. A MGE is considered to be present if at least 70% of its length is covered by reads. Since only very similar sequences are used for the quantification of the contigs, a word length of 15 was chosen for BLASTN searches for practical reasons. Other parameters were referenced from {Stern, 2012 #22} and refined for the quantification of the viral contigs in gut samples.

### The HMP datasets

We downloaded the whole metagenome assemblies (HASM) and gene annotations (HMGI) for the HMP datasets from the DACC website [42].

### Availability of MGE sequences and annotations

We provide the sequences of the MGE contigs for different sets (MGE_all, MGE_nr and MGE_sel) in FASTA files on our MGE website at http://omics.informatics.indiana.edu/mg/MGE. Other information, including the CRISPR spacer/repeat sequences (for MGE identification), protospacer information of the MGE contigs and the annotation of the MGE contigs, is also available at the website.

## Abbreviations

BLAST: Basic Local Alignment Search Tool; BLASTN: Nucleotide Basic Local Alignment Search Tool; bp: base pairs; Cas: CRISPR-associated; COG: clusters of orthologous groups; CRISPR: clustered regularly interspaced short palindromic repeats; GO: Gene Ontology; HMP: Human Microbiome Project; ICE: integrative and conjugative elements; MGE: mobile genetic element; PAM: protospacer-adjacent motif.

## Competing interests

The authors declare that they have no competing interests.

## Author's contributions

QZ carried out the analysis and drafted the manuscript. MR participated in the design and the analysis and helped to draft the manuscript. HT participated in the analysis and helped to draft the manuscript. TGD participated in the analysis and helped to draft the manuscript. YY conceived the study, participated in its design and coordination, participated in the analysis, and helped to draft the manuscript. All authors read and approved the final manuscript.

## Supplementary Material

Additional file 1 **Figure S1-3 and Tables S1-2**. Click here for file

Additional file 2 **Spreadsheets of significantly enriched or depleted GO terms**. Click here for file

Additional file 3 **A list of proteins annotated as associated with bacterial restriction-modification systems**. Click here for file
