# Supplementary material for: CRISPR-Cas systems target a diverse collection of invasive mobile genetic elements in human microbiomes
Source: Genome Biol. 2013 Apr 29;14(4):R40. doi: 10.1186/gb-2013-14-4-r40 (PMC4053933; doi:10.1186/gb-2013-14-4-r40)
Supplement: Additional file 1 — Figure S1-3 and Tables S1-2. [file gb-2013-14-4-r40-S1.PDF]

**This document contains Supplementary Figs. 1–3 and Supplementary Tables 1–2**

## CRISPR–Cas systems target a diverse collection of invasive mobile genetic elements in human microbiomes

Quan Zhang[1#], Mina Rho[2#], Haixu Tang[13], Thomas G. Doak[4], Yuzhen Ye[1§]

A

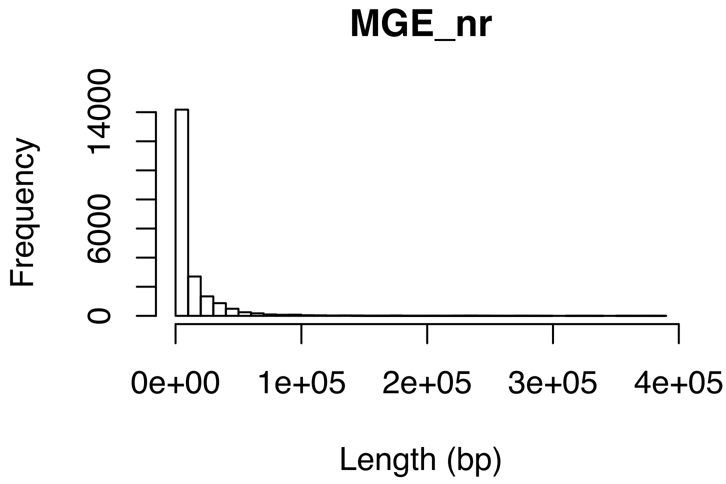


B

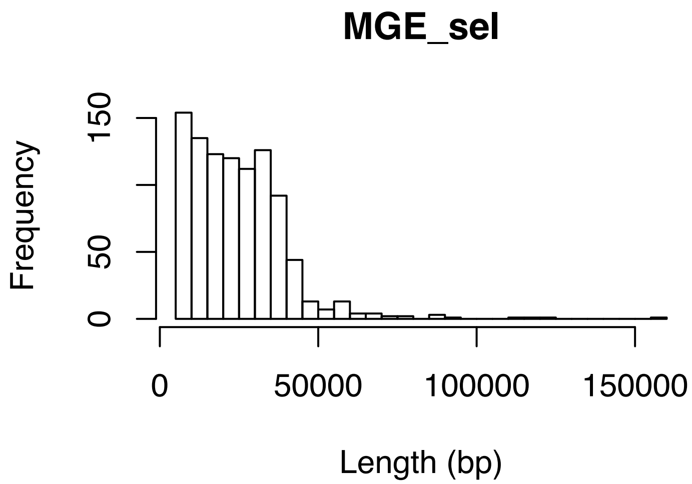


**Supplementary Figure 1**. The length distribution of the MGE contigs in the MGE_nr set (A) and the MGE segments with dense proto-spacers (B).

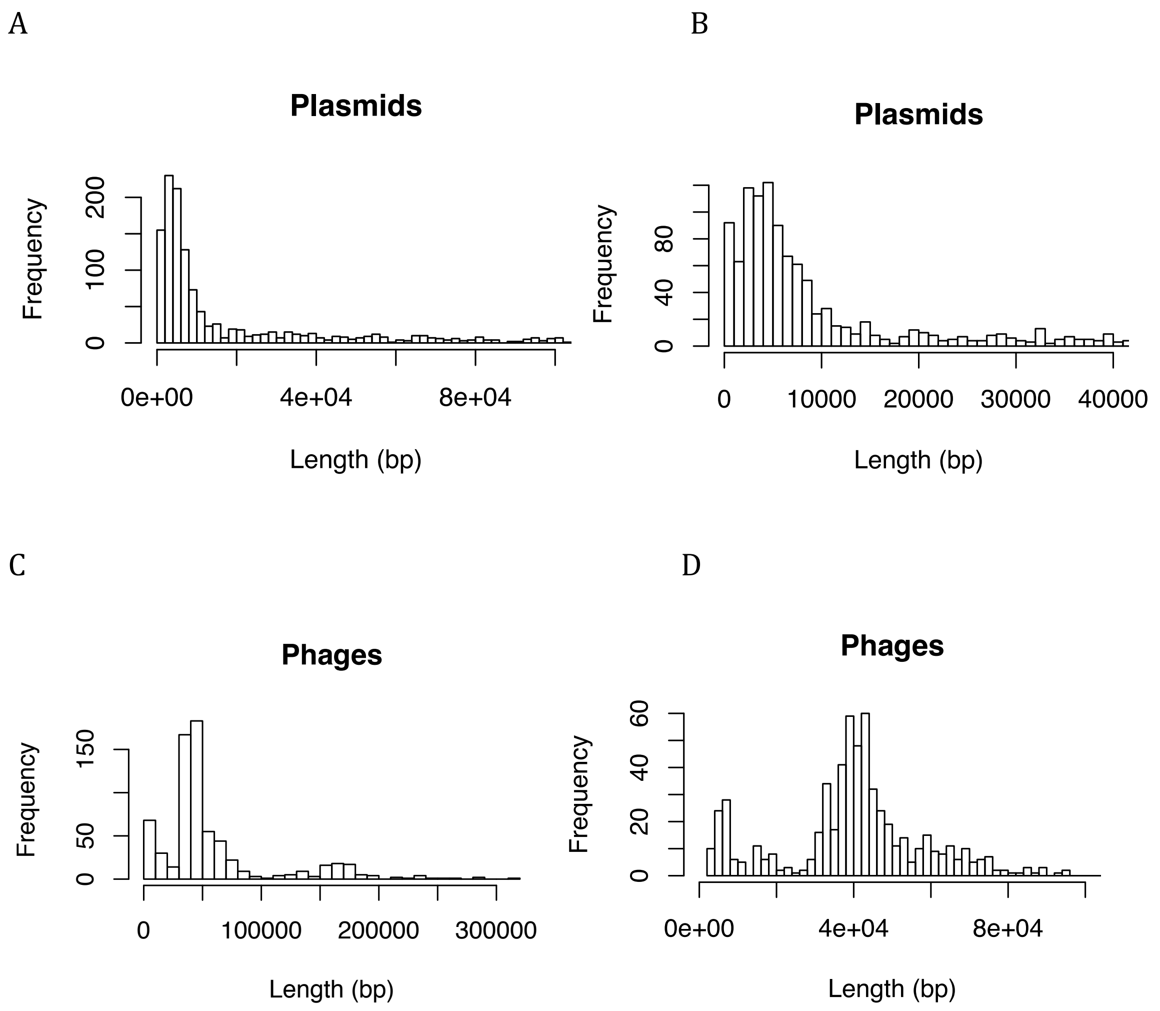


**Supplementary Figure 2**. The length distribution of plasmid (A) (zoomed in with maximum length set to 40000 in figure B) the and phage (B) (zoomed in with maximum length set to 100000 in figure D) genomes.

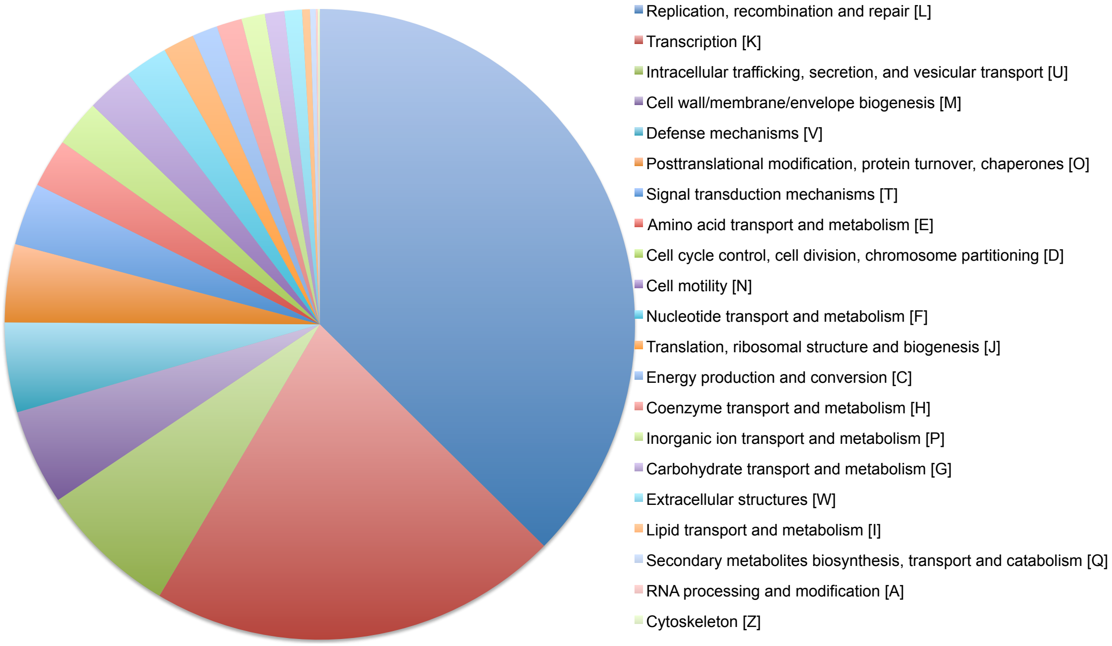


**Supplementary Figure 3.** COG distribution of the proteins encoded by the MGE contigs.

**Supplementary Table 1**. The abundance plots of selected MGEs (total 98) and their putative bacterial hosts. The MGEs are grouped into three categories (see texts for details).

## Category I

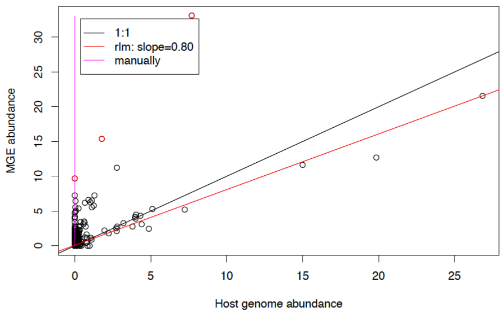


SRS019126_WUGC_scaffold_42151

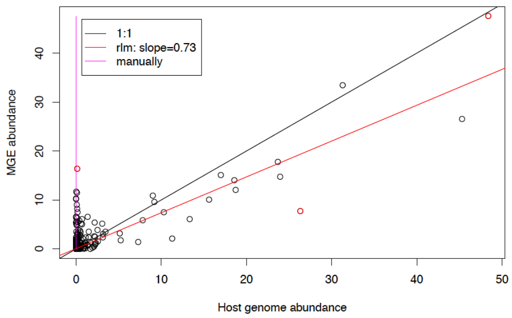


SRS022532_LANL_scaffold_5284

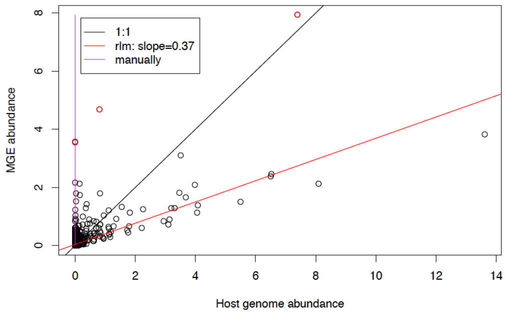


SRS022536_LANL_scaffold_112417

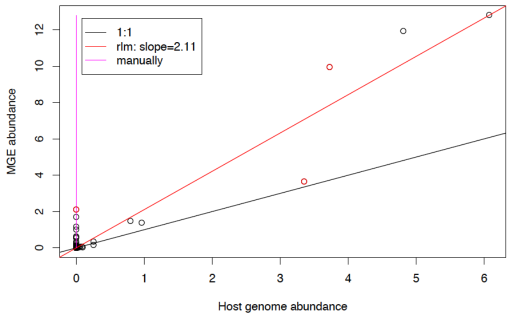


SRS048791_LANL_scaffold_63004

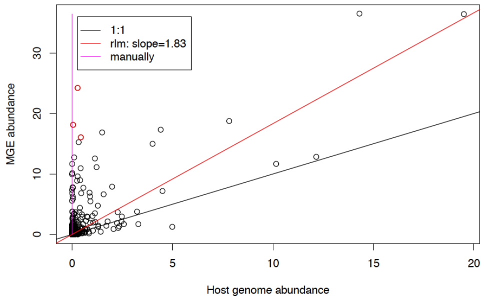


SRS054569_LANL_scaffold_2376

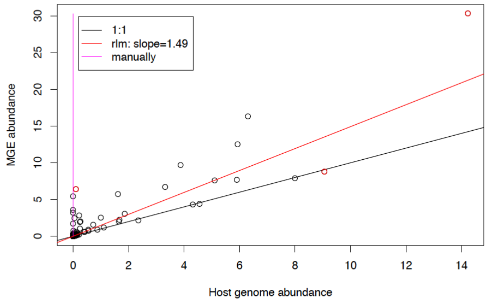


SRS018145_Baylor_scaffold_29251

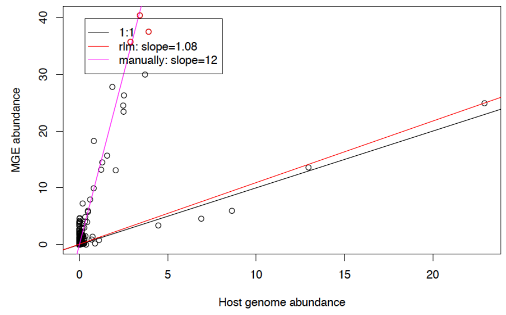


SRS064329_LANL_scaffold_35322

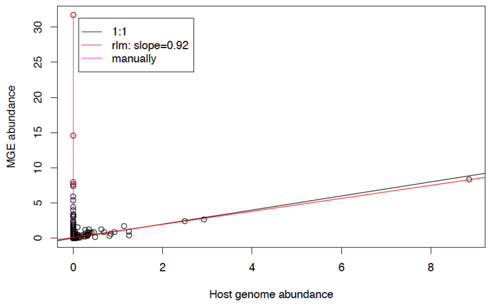


SRS044662_LANL_scaffold_46036

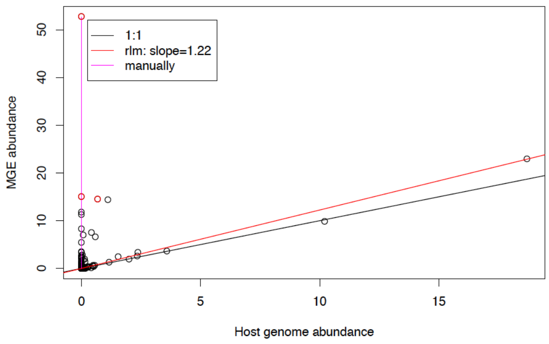


SRS045715_LANL_scaffold_59715

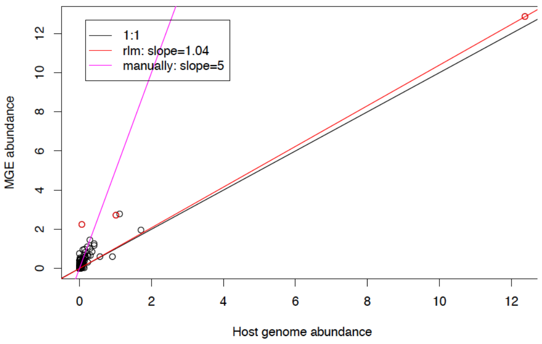


SRS058808_LANL_scaffold_23764

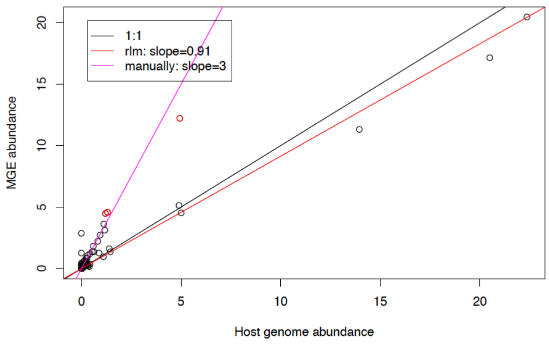


SRS016002_WUGC_scaffold_61510

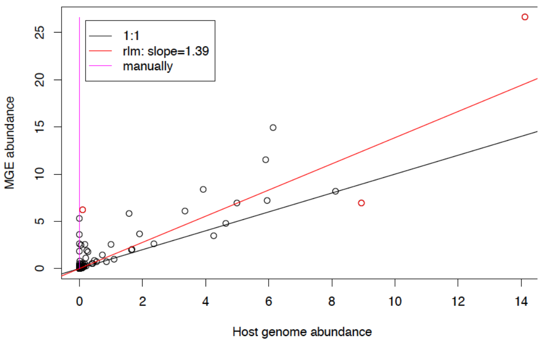


SRS052227_LANL_scaffold_8852

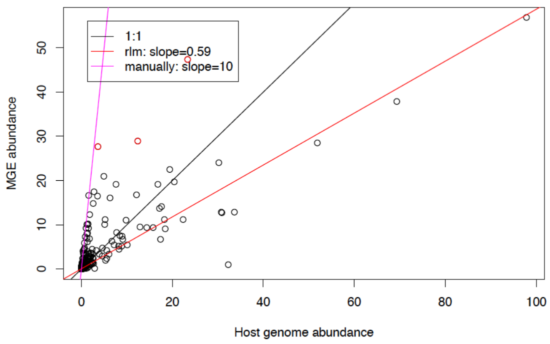


SRS015378_WUGC_scaffold_9976

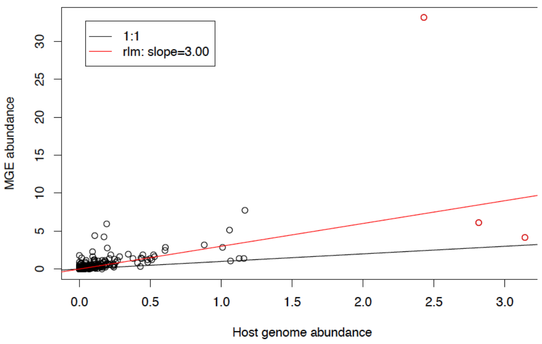


SRS019607_WUGC_scaffold_56123

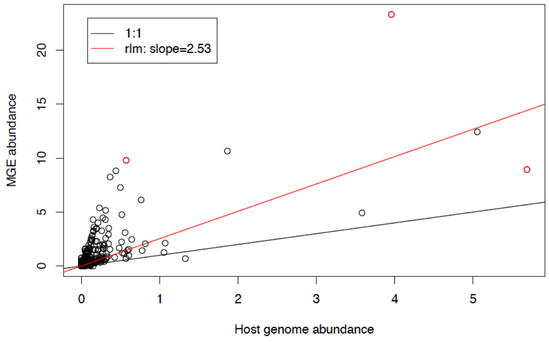


SRS044373_WUGC_scaffold_65460

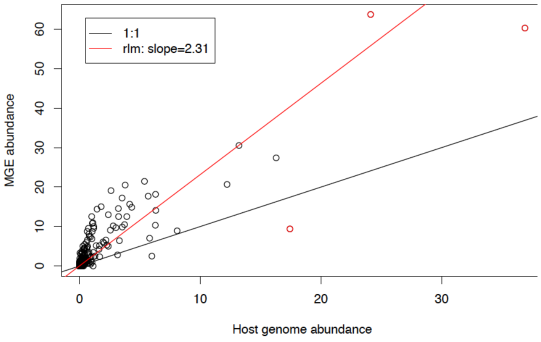


SRS020862_Baylor_scaffold_11192

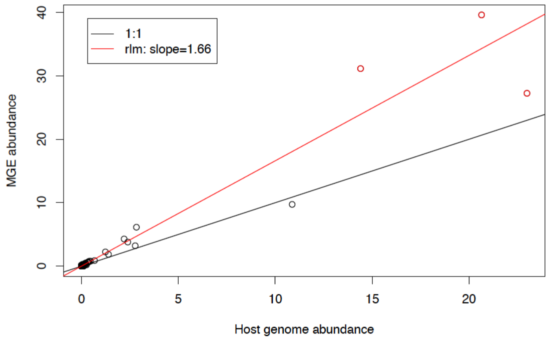


SRS019028_WUGC_scaffold_10379

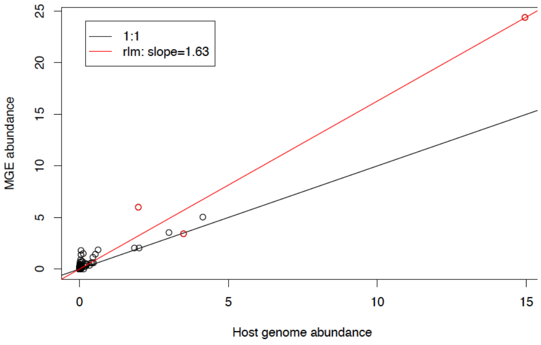


SRS063193_LANL_scaffold_66015

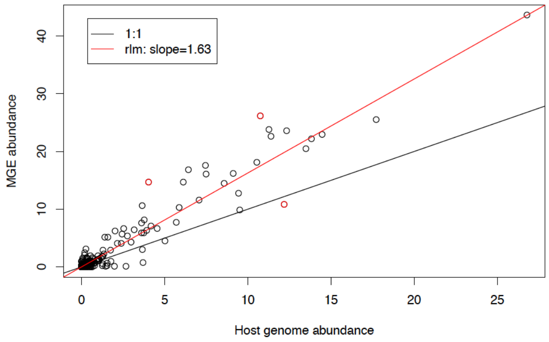


SRS013164_Baylor_scaffold_4275

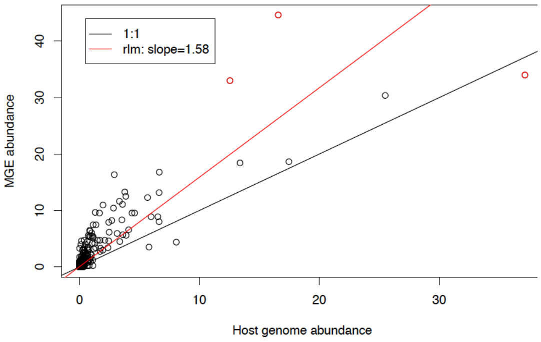


SRS020340_Baylor_scaffold_3253

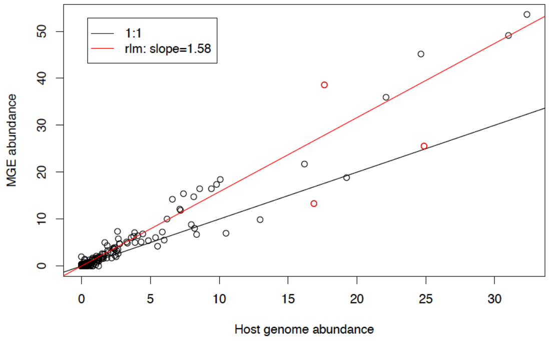


SRS016039_WUGC_scaffold_16219

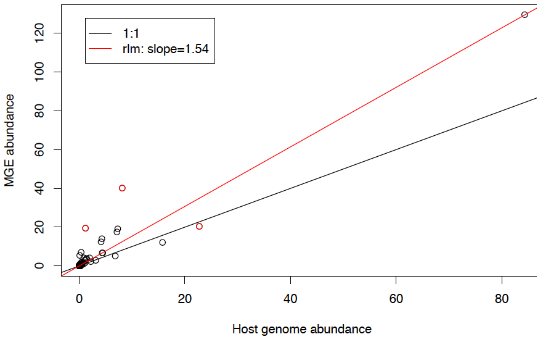


SRS018439_Baylor_scaffold_62023

Category II

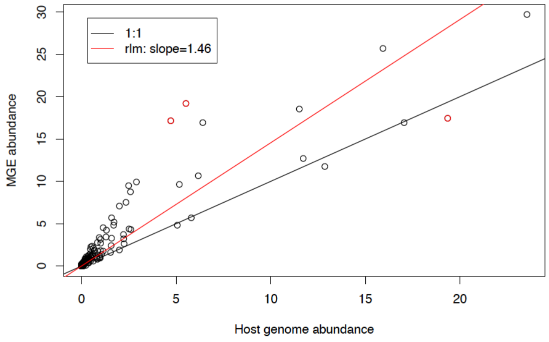


SRS051791_LANL_scaffold_33739

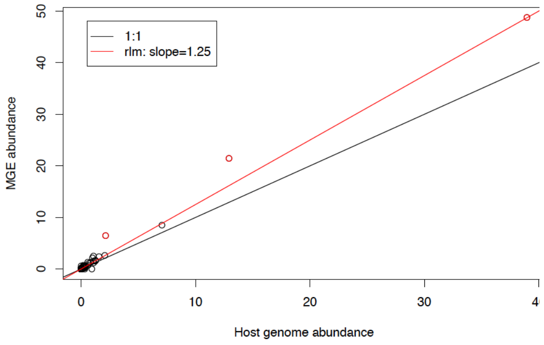


SRS018300_Baylor_scaffold_18966

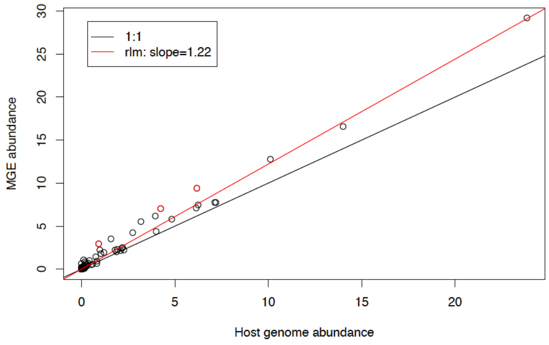


SRS022530_LANL_scaffold_21325

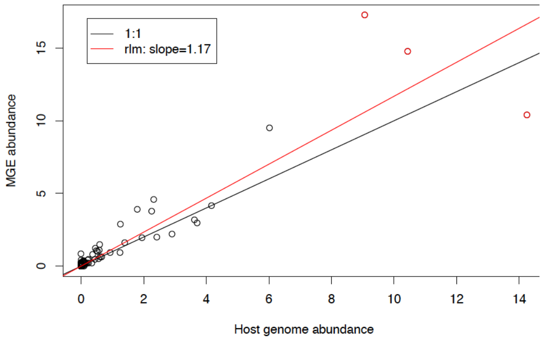


SRS057205_LANL_scaffold_59215

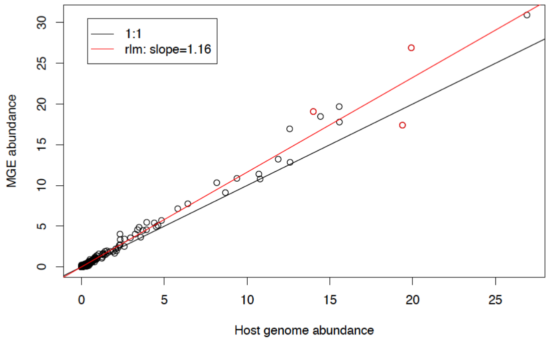


SRS053854_LANL_scaffold_26921

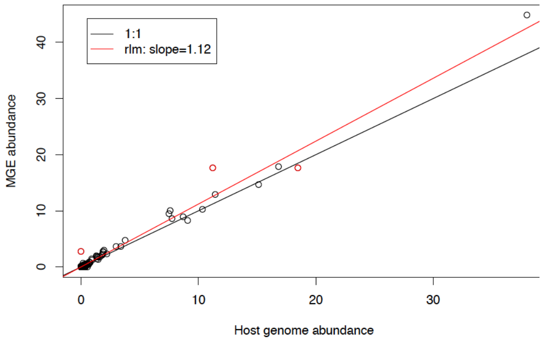


SRS015057_WUGC_scaffold_902

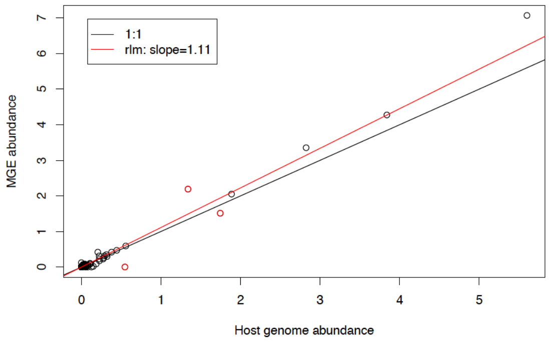


SRS015941_WUGC_scaffold_2450

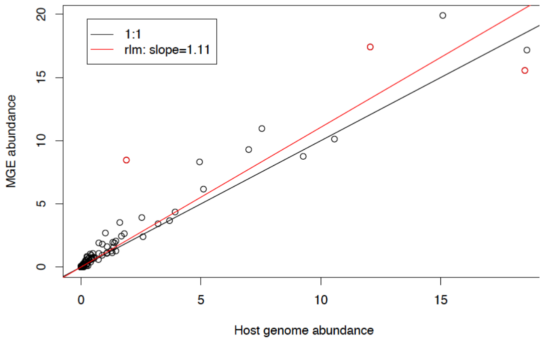


SRS058336_LANL_scaffold_3483

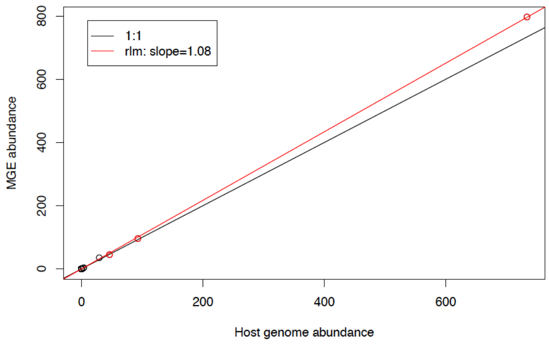


SRS015755_WUGC_scaffold_47054

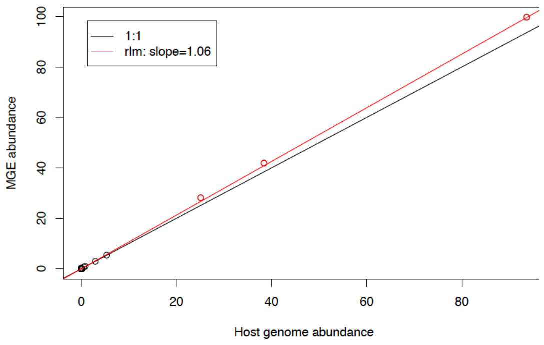


SRS017691_Baylor_scaffold_77658

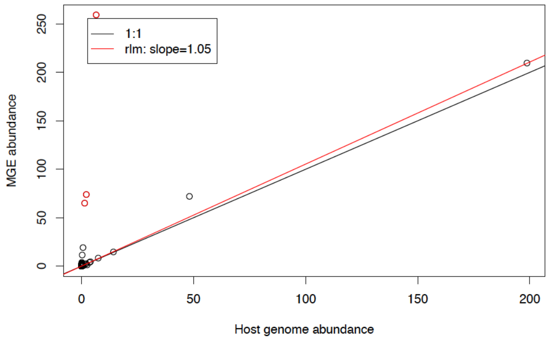


SRS019391_WUGC_scaffold_2239

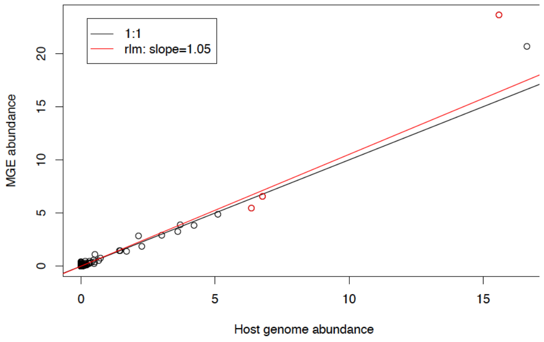


SRS015941_WUGC_scaffold_15301

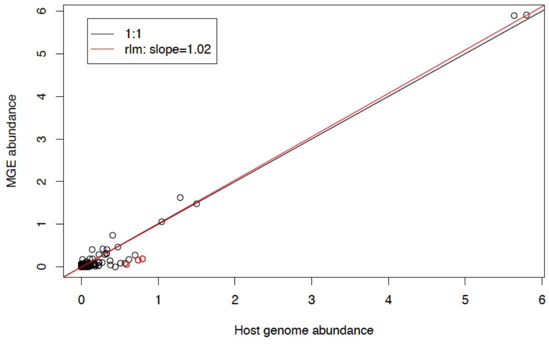


SRS052227_LANL_scaffold_38054

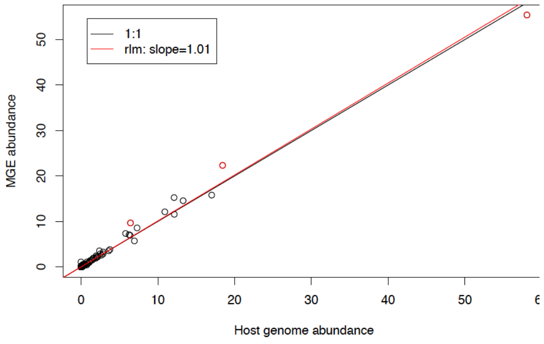


SRS018591_WUGC_scaffold_4777

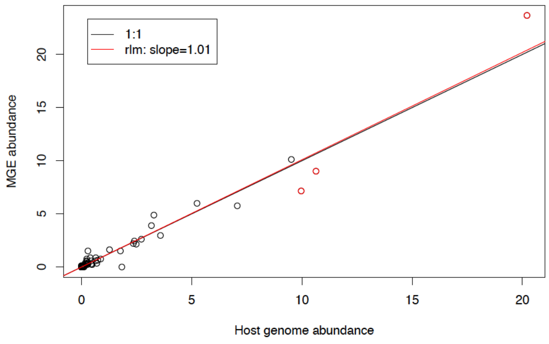


SRS015762_WUGC_scaffold_51515

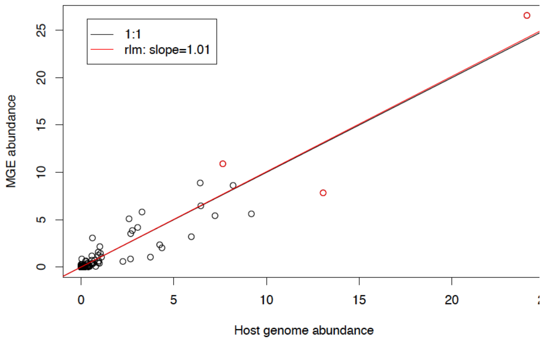


SRS024081_LANL_scaffold_73425

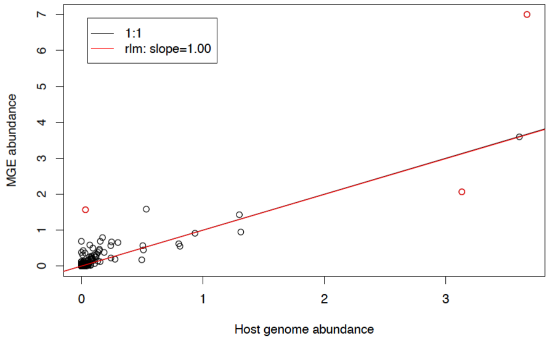


SRS049318_LANL_scaffold_28254

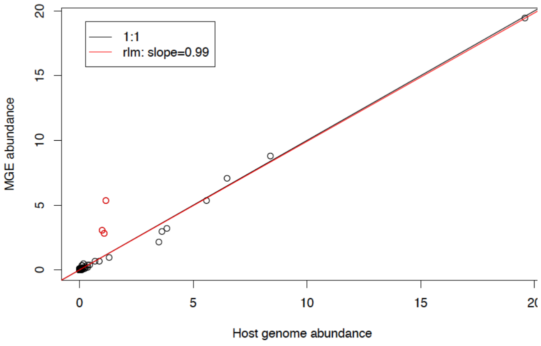


SRS057539_LANL_scaffold_13064

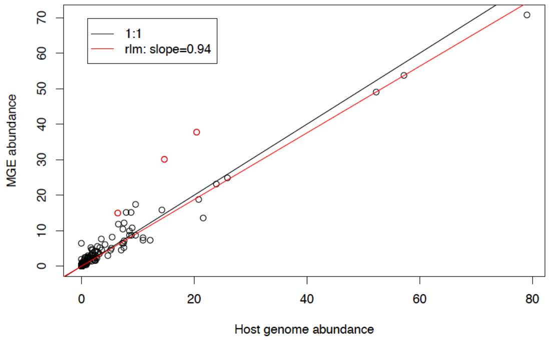


SRS019045_WUGC_scaffold_15733

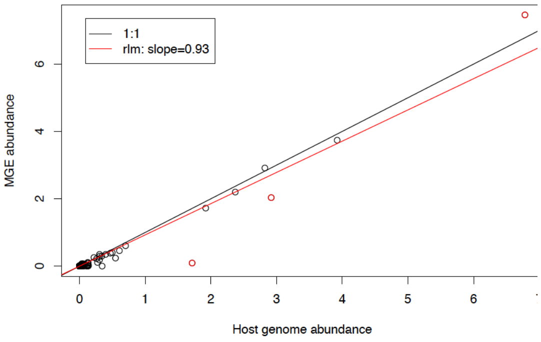


SRS053603.C3333952

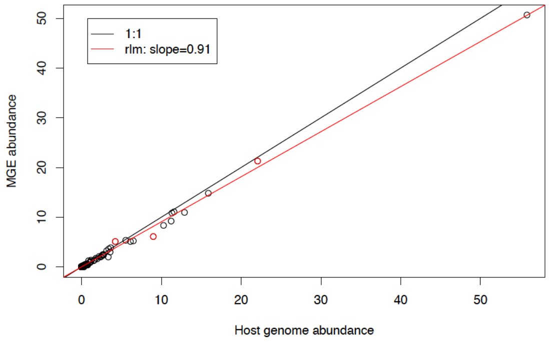


SRS051791_LANL_scaffold_3222

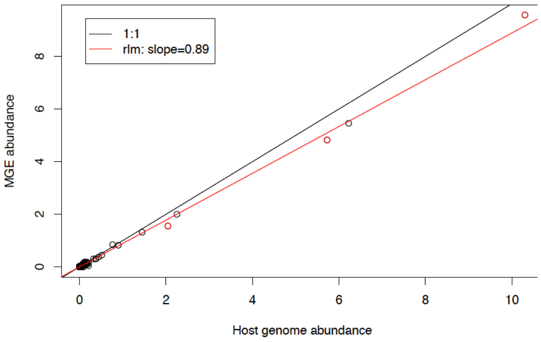


SRS024277_LANL_scaffold_64593

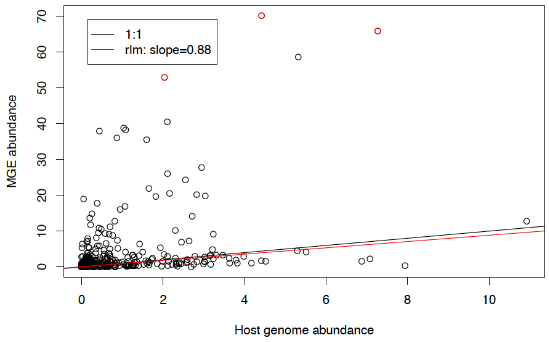


SRS051791_LANL_scaffold_7216

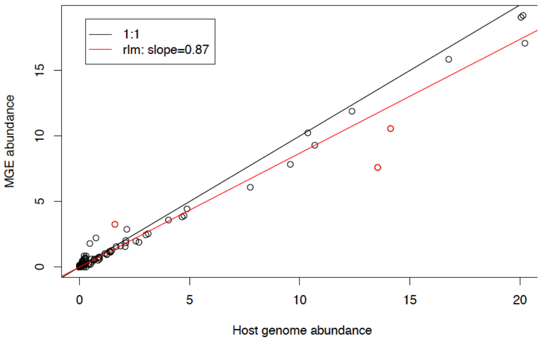


SRS022530_LANL_scaffold_56387

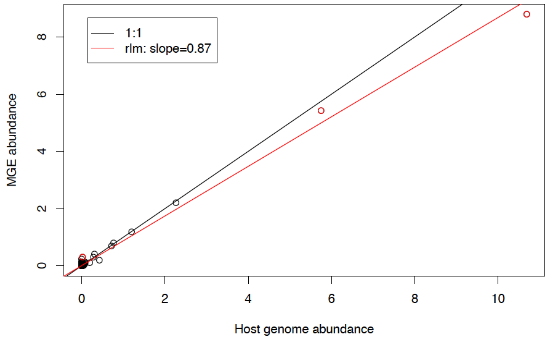


SRS062761_LANL_scaffold_38752

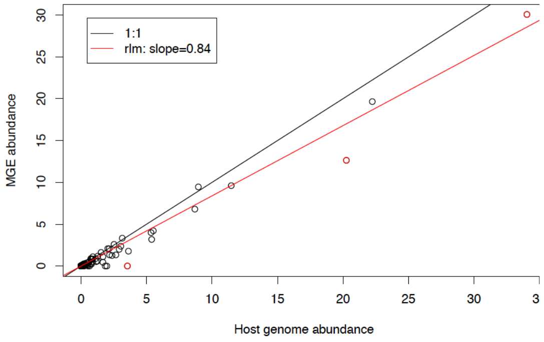


SRS019327_WUGC_scaffold_71581

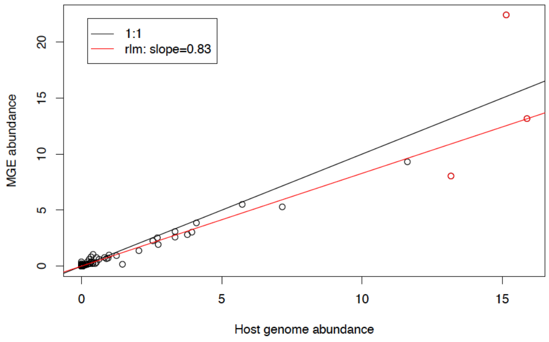


SRS019327_WUGC_scaffold_35742

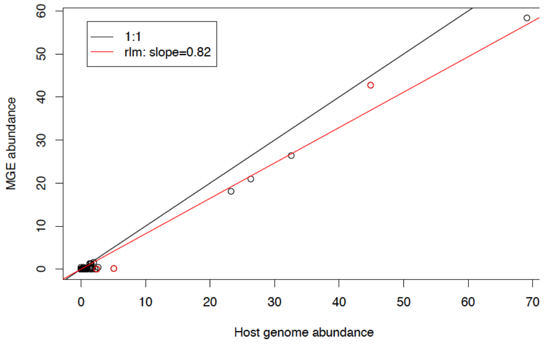


SRS014474.C734144

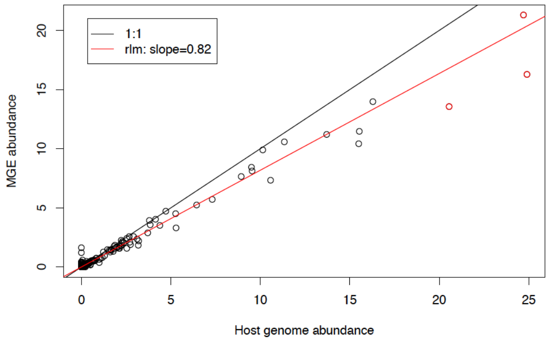


SRS053854_LANL_scaffold_20248

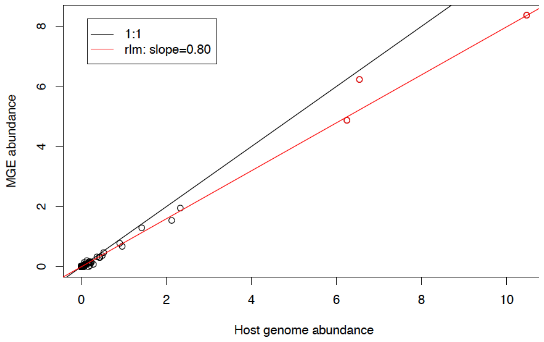


SRS018357_Baylor_scaffold_53355

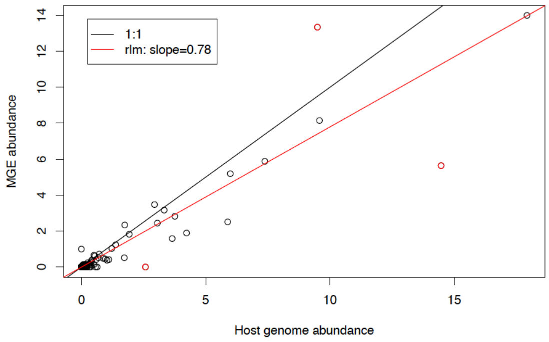


SRS042643_WUGC_scaffold_24503

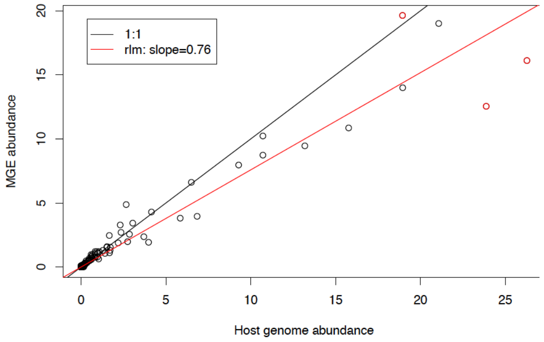


SRS016037_WUGC_scaffold_3155

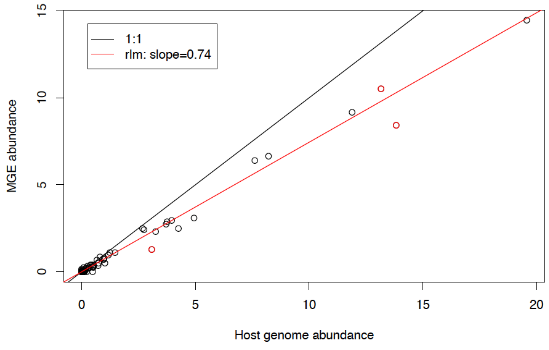


SRS042643_WUGC_scaffold_65762

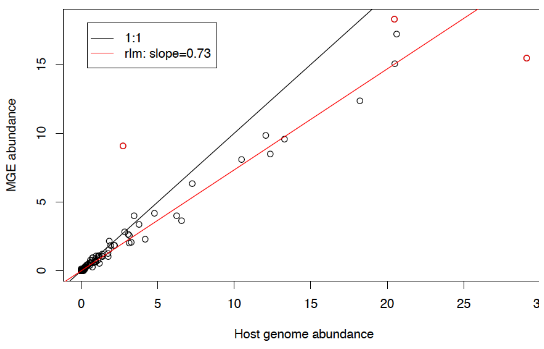


SRS022621_Baylor_scaffold_720

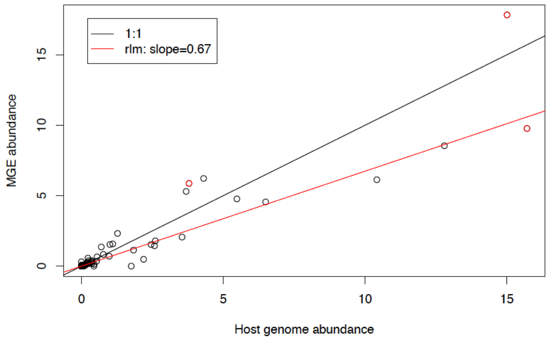


SRS019027.C1988766

## Category III

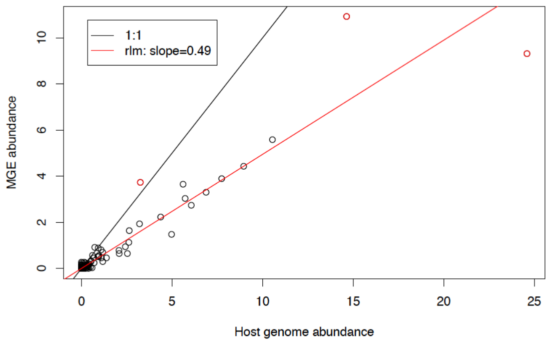


SRS057791_LANL_scaffold_34998

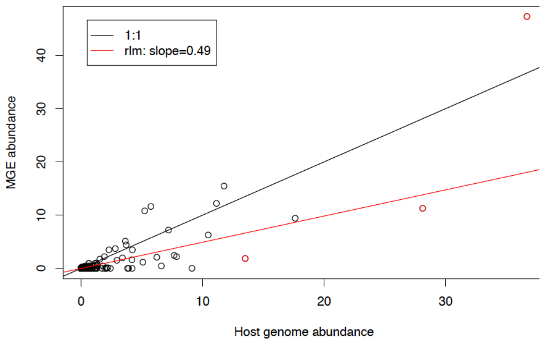


SRS064423_LANL_scaffold_5820

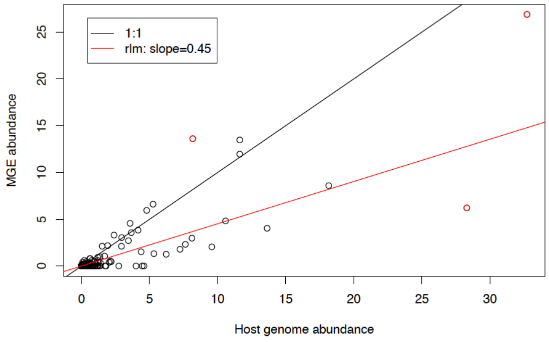


SRS047824_WUGC_scaffold_46520

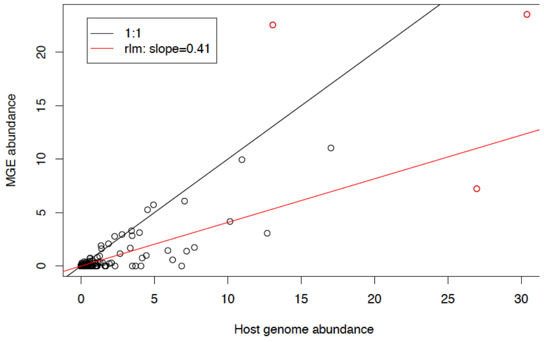


SRS022530_LANL_scaffold_32610

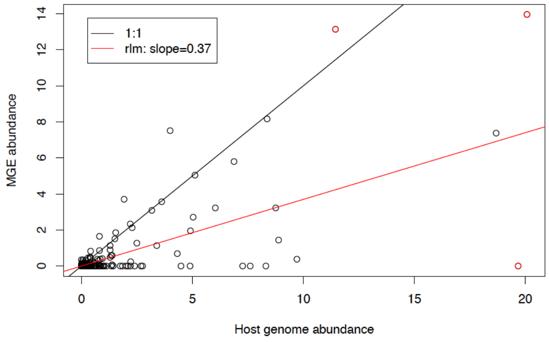


SRS044662.C1686457

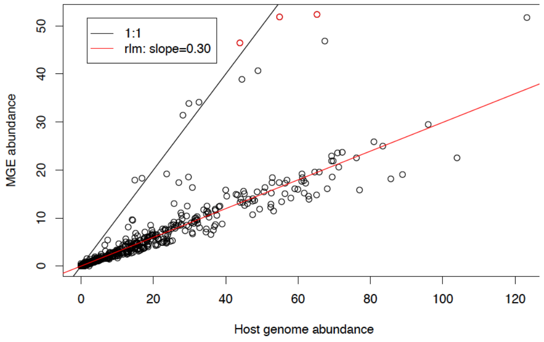


SRS016092_WUGC_scaffold_7184

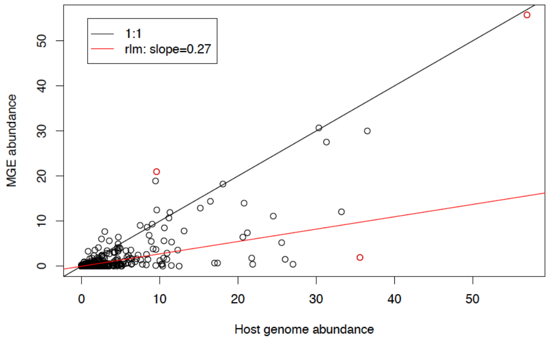


SRS024377_LANL_scaffold_134

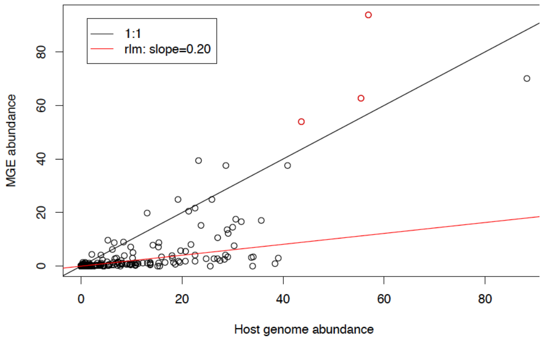


SRS024144_LANL_scaffold_25869

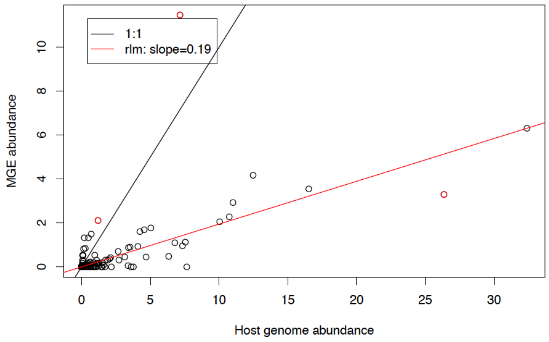


SRS012279_Baylor_scaffold_48257

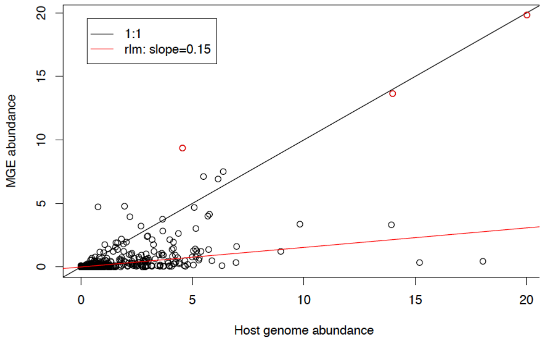


SRS053630_LANL_scaffold_370

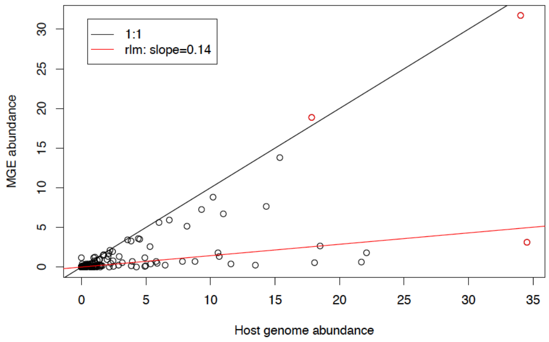


SRS053854_LANL_scaffold_51880

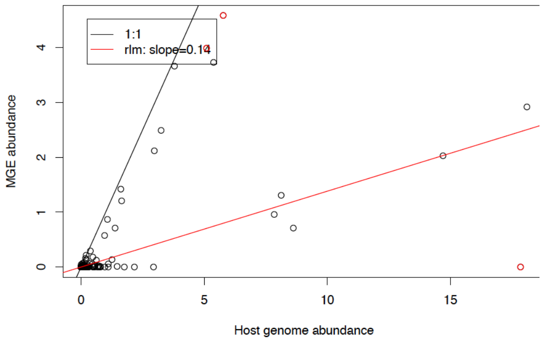


SRS018573_WUGC_scaffold_41588

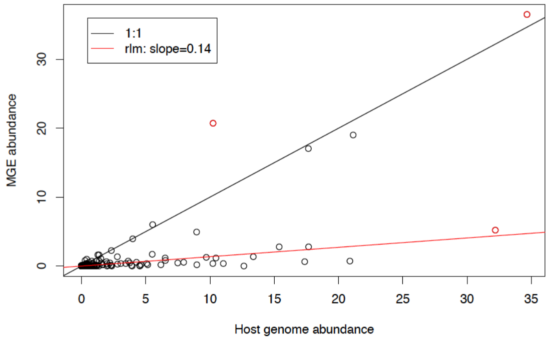


SRS064329_LANL_scaffold_51057

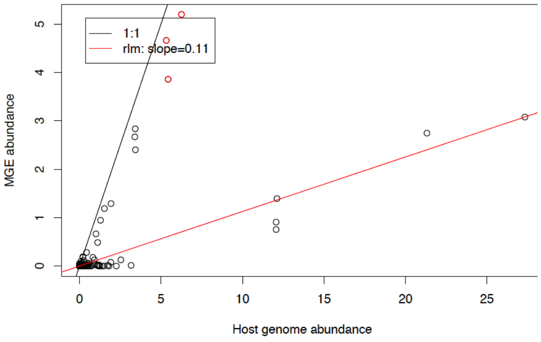


SRS024447_LANL_scaffold_59616

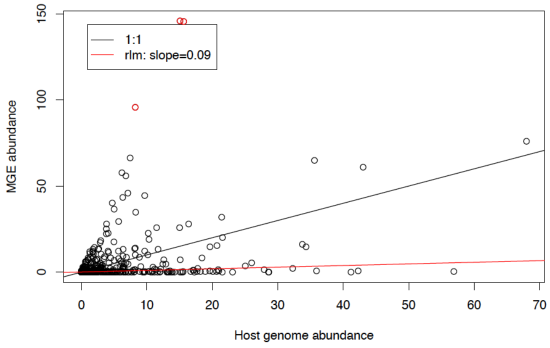


SRS022530_LANL_scaffold_7588

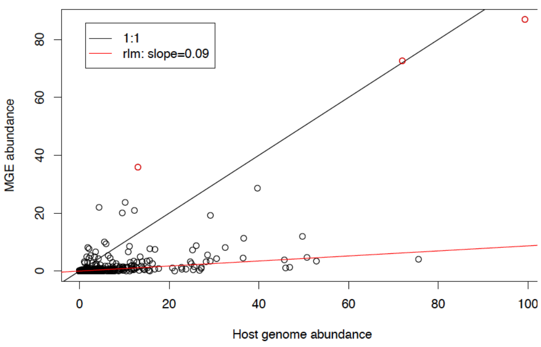


SRS015985_WUGC_scaffold_35939

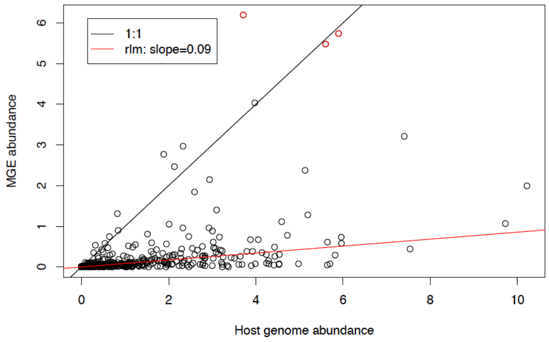


SRS022530_LANL_scaffold_6824

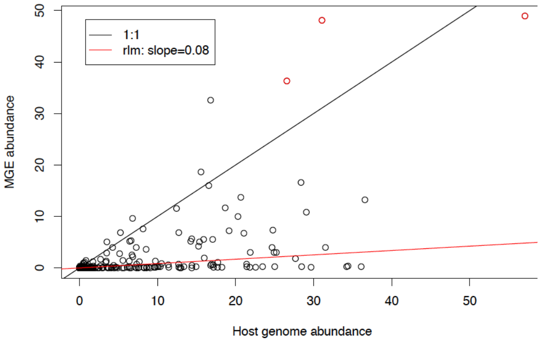


SRS016043_WUGC_scaffold_8228

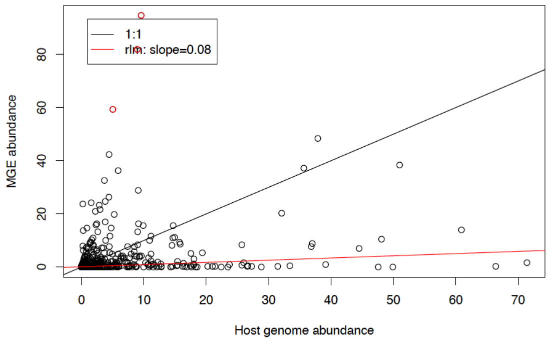


SRS016200_WUGC_scaffold_12207

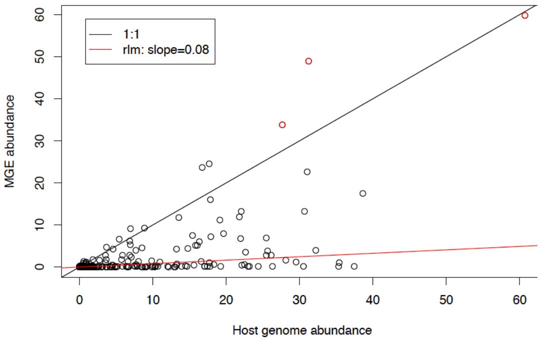


SRS016575_Baylor_scaffold_2114

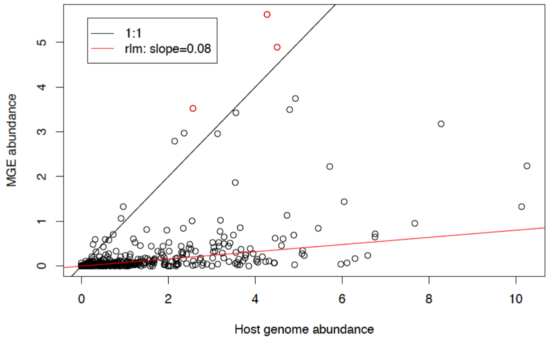


SRS064423_LANL_scaffold_55363

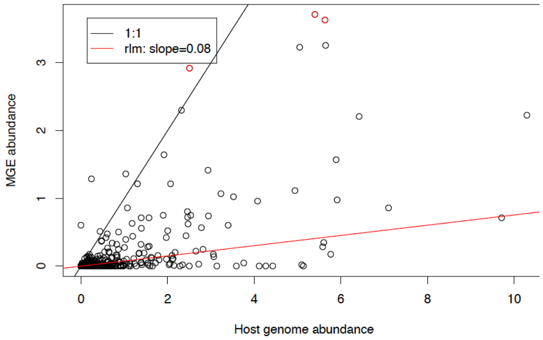


SRS017209_Baylor_scaffold_66158

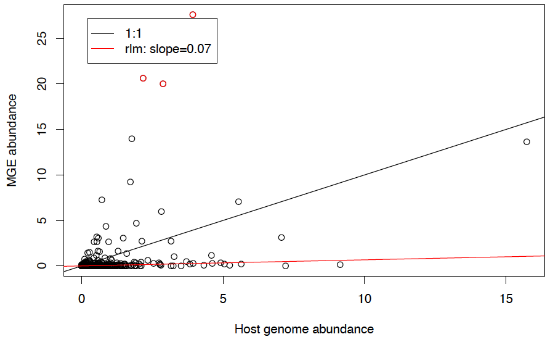


SRS019980_Baylor_scaffold_18220

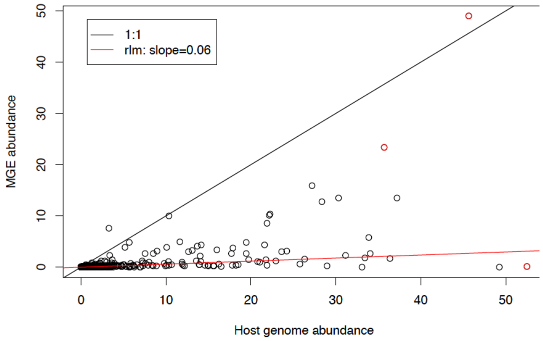


SRS024140_LANL_scaffold_4000

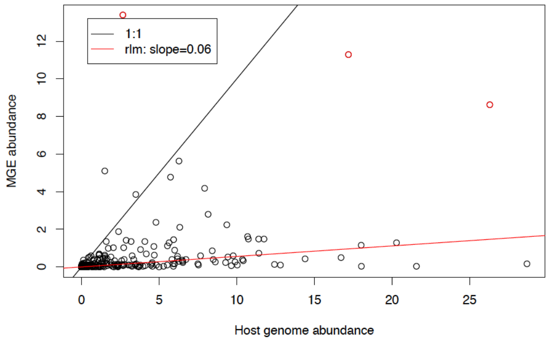


SRS016569_Baylor_scaffold_7554

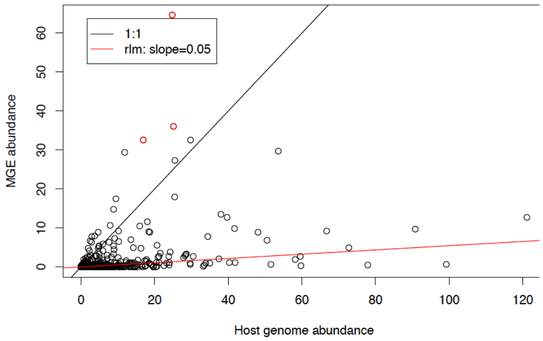


SRS020862_Baylor_scaffold_11605

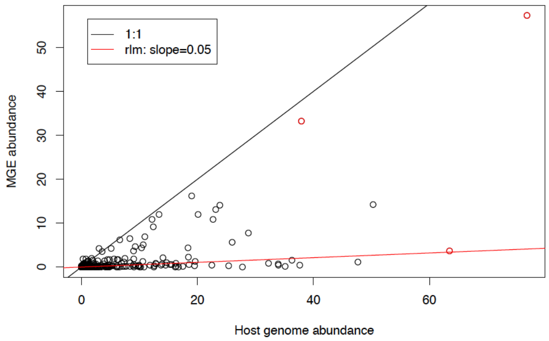


SRS057791_LANL_scaffold_81805

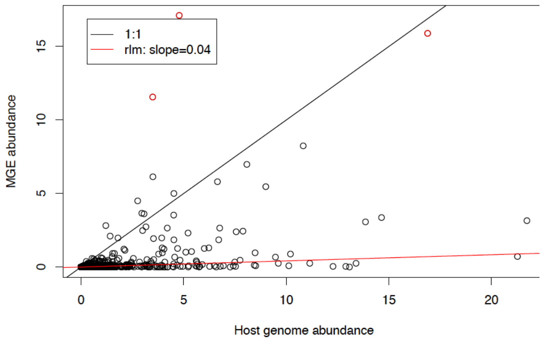


SRS016002_WUGC_scaffold_521

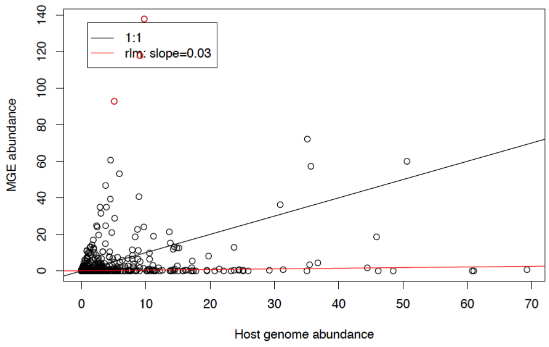


SRS016092_WUGC_scaffold_1002

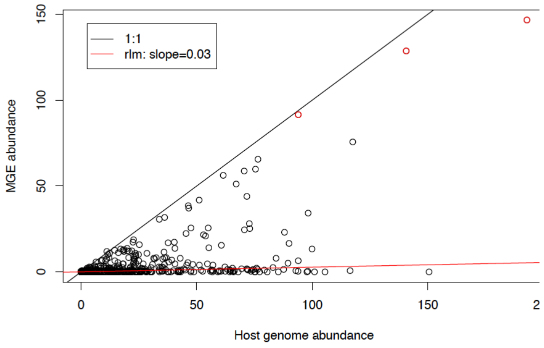


SRS015215_WUGC_scaffold_55448

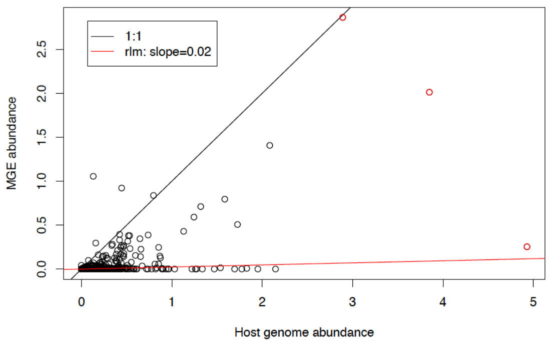


SRS022725_LANL_scaffold_20366

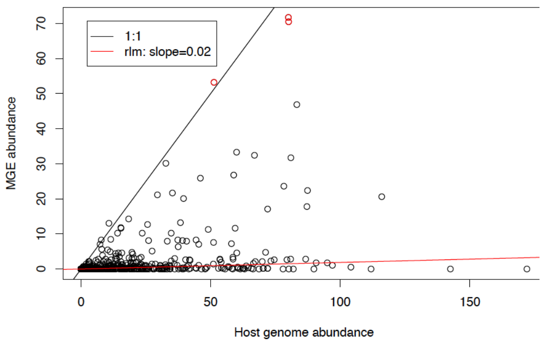


SRS053630_LANL_scaffold_2818

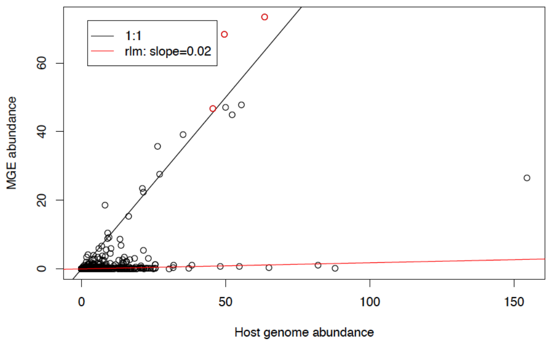


SRS013881_WUGC_scaffold_945

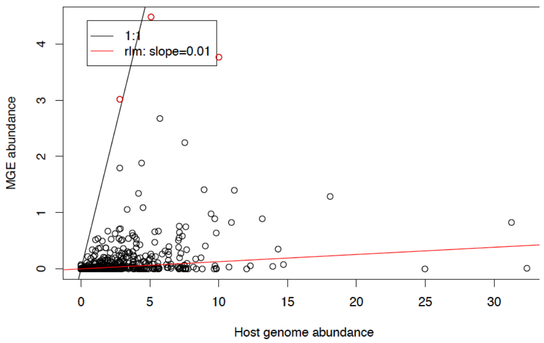


SRS013252_Baylor_scaffold_65537

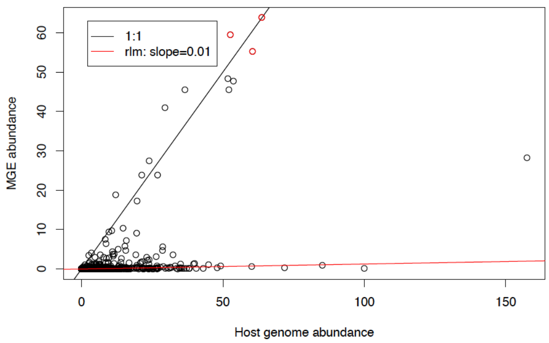


SRS022536_LANL_scaffold_98123

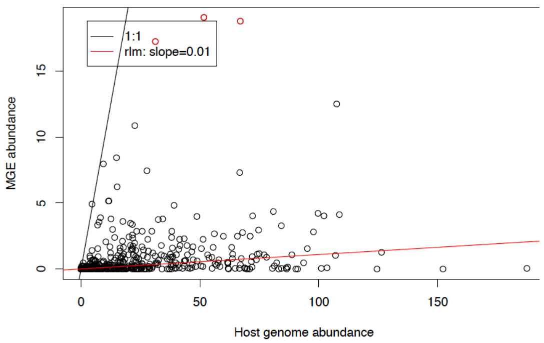


SRS023595_Baylor_scaffold_21679

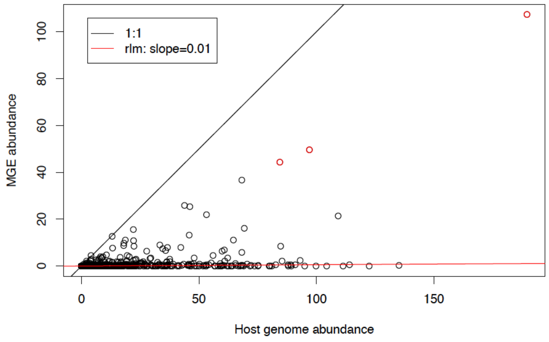


SRS017304_Baylor_scaffold_27680

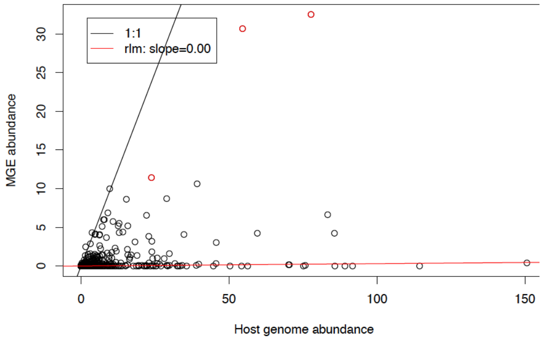


SRS023617_Baylor_scaffold_6414

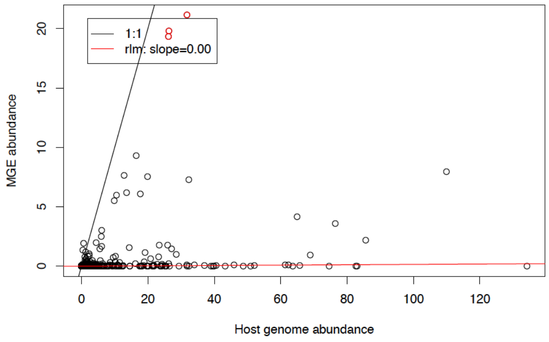


SRS015644_WUGC_scaffold_4581

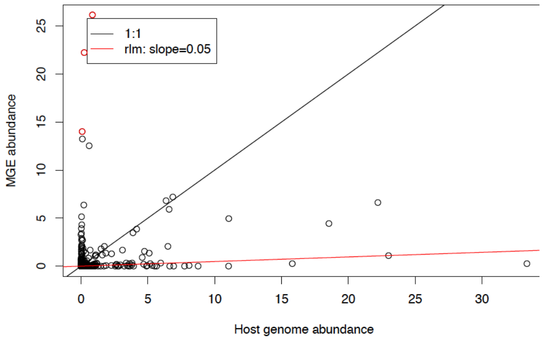


SRS021477_Baylor_scaffold_56906

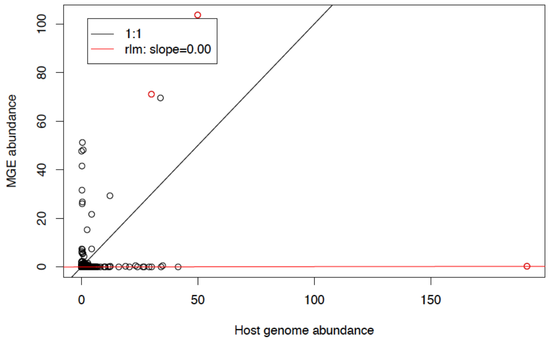


SRS021496_Baylor_scaffold_9811

**Supplementary Table 2.** Example MGEs that lack PAMs.

| CRISPR type | MGE | | |
|---|---|---|---|
| | ID | #(protospacer) | Bit* |
| SRS062761L37 ( crispr85) | SRS052227_LANL_scaffold_29813 | 90 | 0.06 |
| SRS014470L37 ( crispr94) | SRS019327_WUGC_scaffold_71581 | 96 | 0.08 |
| Veil_sp3_1_44_L36 ( crispr131) | SRS015762_WUGC_scaffold_43987 | 125 | 0.12 |
| SRS012279L36 ( crispr87) | SRS022143_WUGC_scaffold_24879 | 250 | 0.17 |
| Prev_t472_L36 ( crispr181) | SRS049318_LANL_scaffold_185700 | 62 | 0.19 |
| SRS018394L37 ( crispr53) | SRS022530_LANL_scaffold_21325 | 244 | 0.19 |
| SRS056323L36 ( crispr93) | SRS018439_Baylor_scaffold_27954 | 149 | 0.20 |
| SRS013506L37 ( crispr14) | SRS022530_LANL_scaffold_2162 | 32 | 0.21 |
| SRS011152L36 ( crispr59) | SRS057205_LANL_scaffold_49165 | 156 | 0.27 |
| Veil_sp3_1_44_L35 ( crispr132) | SRS048791_LANL_scaffold_63004 | 59 | 0.28 |
| Prev_t317_L37 ( crispr179) | SRS019028_WUGC_scaffold_45396 | 23 | 0.29 |
| SRS018300L36 ( crispr89) | SRS047219_WUGC_scaffold_61852 | 41 | 0.36 |
| SRS019127L37 ( crispr79) | SRS018439_Baylor_scaffold_61485 | 30 | 0.36 |

*: The conservation score of the most conserved position in the adjacent regions of the protospacers.
